# Shen-Ling-Bai-Zhu-San Mitigates Ulcerative Colitis by Enhancing Intestinal Barrier Integrity *via* the AhR-CYP1A1-NF-κB Signal Pathway

**DOI:** 10.2174/0113862073425771250828112001

**Published:** 2025-09-30

**Authors:** Lan Ming, Jie Chen, Jing Ma, ShiQi Guo, Ke Xu, JiaMin Ji, ZhiRong Zhao, ShuGuang Xu, Qian Huang

**Affiliations:** 1 YanchengTCM Hospital Affiliated to Nanjing University of Chinese Medicine, Yancheng 224000, China;; 2 Jinling Clinical Medical College, Nanjing University of Chinese Medicine, Nanjing 210000, China;; 3 Research Institute of General Surgery, Jinling Hospital, Southeast University, Nanjing 210000, China;; 4 Research Institute of General Surgery, Jinling Hospital, Nanjing University, Nanjing 210000, China

**Keywords:** AhR, CYP1A1, mechanism, shen-ling-bai-zhu-san, intestinal barrier, ulcerative colitis

## Abstract

**Introduction:**

Ulcerative Colitis (UC) represents a persistent inflammatory disorder of the colon, typically characterized by abdominal discomfort, diarrhea, and blood stools. Shen-Ling-Bai-Zhu-San (SLBZS), a traditional Chinese herbal formula, has shown clinical efficacy in alleviating symptoms such as abdominal bloating, frequent loose stools, and diarrhea. Nonetheless, the precise molecular mechanisms underlying its therapeutic effects remain largely unclear.

**Methods:**

UPLC-QE-MS combined with network pharmacology was employed to identify bioactive compounds and potential targets of SLBZS. A Dextran Sulfate Sodium (DSS)-induced colitis mouse model was used to evaluate its effects by monitoring changes in body weight, colon length, Disease Activity Index (DAI), inflammatory cytokines, oxidative stress markers, tight junction proteins, immunofluorescence, and histopathology. Molecular docking was used to predict the interaction of active compounds with UC-related targets, and Western blot analysis was performed to validate signaling pathways.

**Results:**

SLBZS markedly improved DSS-induced colitis by restoring body weight, colon length, DAI, and histology. It suppressed pro-inflammatory cytokines and oxidative markers while enhancing antioxidant defenses. Expression of Occludin and Claudin-1 was recovered. UPLC-MS/MS identified 458 constituents, and network pharmacology revealed 98 potential targets enriched in NF-κB, TNF, and HIF-1 pathways. Validation experiments demonstrated the upregulation of AhR and CYP1A1 with concomitant downregulation of NLRP3 and IL-6. Molecular docking confirmed high-affinity interactions between key compounds and UC-related targets.

**Discussion:**

These results indicate that SLBZS exerts its effects through anti-inflammatory and antioxidant mechanisms while strengthening the intestinal barrier, reflecting its multi-target therapeutic potential.

**Conclusions:**

SLBZS alleviates UC by regulating the AhR-CYP1A1-NF-κB axis, suppressing inflammation, and maintaining mucosal barrier function.

## 1 INTRODUCTION

Ulcerative Colitis (UC) is a major subtype of inflammatory bowel disease that significantly impairs patient quality of life [1]. Current evidence attributes its increasing prevalence to environmental factors, intestinal barrier dysfunction, immune dysregulation, and alterations in gut microbiota composition [2]. Disruption of the intestinal barrier is a hallmark of UC [3]. This barrier is primarily formed by Tight Junctions (TJs) between epithelial cells, which serve as the first line of defence against pathogenic invasion [4]. TJs comprising proteins, such as occludins, ZO-1, junctional adhesion molecules, and cingulin, restrict the passage of harmful substances and microorganisms across the epithelium [5], thereby regulating mucosal permeability [6]. In both UC patients and experimental colitis models, compromised barrier integrity and reduced TJ protein levels are frequently observed, contributing to excessive mucosal permeability [7]. Thus, restoration of the damaged intestinal barrier is a critical therapeutic goal in UC.

Current standard treatments include aminosalicylates, glucocorticoids, biologics, and enteral nutrition [8, 9]. However, these interventions often produce notable side effects, limiting their long-term use. Nanomedicine-based approaches, such as drug nanocrystals, have emerged as promising strategies to enhance therapeutic delivery and efficacy [10, 11]. Recently, Traditional Chinese Medicine (TCM) has garnered growing interest from clinicians due to its notable effectiveness and minimal toxicity.

First developed during the Song Dynasty in China, Shen-Ling-Bai-Zhu-San (SLBZS) possesses a significant historical background and is officially acknowledged in the pharmacopoeia. Its composition includes *Codonopsis pilosula (Franch.) Nannf*, *Poria cocos (Schw.) Wolf*, *Atractylodes macrocephala Koidz*, *Dioscorea opposite Thunb.*, *Dolichos Lablab L.*, *Semen Nelumbinis.*, *Glycyrrhiza uralensis Fisch.*, *Amomum villosum Lour.*, *Coix lacryma-jobi L.*, and *Platycodon grandifloras* [12, 13]. SLBZS is a well-established herbal prescription historically employed to manage diarrhea associated with spleen deficiency. In the context of TCM, UC is categorized under conditions such as “hematochezia” and “loose stools”, with impaired spleen function considered a fundamental pathogenic factor. Recent studies have shown SLBZS’s efficacy against UC in alleviating symptoms like abdominal discomfort, diarrhea, and hematochezia. Clinical evidence suggests that SLBZS enhances efficacy, reduces inflammation, and minimizes side effects compared to traditional medications [14, 15]. Moreover, SLBZS has demonstrated efficacy in alleviating the symptoms of UC in animal models induced by treatment with Dextran Sulfate Sodium (DSS), while also increasing beneficial gut microbiota and modulating intestinal microbiota [16, 17]. Additionally, some SLBZS components act as ligands for the Aryl hydrocarbon Receptor (AhR) [18], a ligand-activated transcription factor involved in diverse physiological and pathological processes [19, 20]. AhR regulates the expression of CYP1A1, a cytochrome P450 enzyme that plays a crucial role in inflammatory modulation [21, 22].

Preclinical studies suggest that AhR activation can suppress NF-κB signaling, enhance epithelial tight junction expression, and mitigate mucosal inflammation [23]. In UC, NF-κB is frequently overactivated, leading to persistent immune imbalance [24]. At the molecular level [25], AhR is capable of detecting phytochemicals and plays a role in maintaining mucosal immune equilibrium, while its downstream effector, CYP1A1, is implicated in the control of inflammatory lipid pathways. NF-κB, a well-characterized transcriptional regulator of inflammation, is subject to modulation by AhR activity. Although AhR ligands have been reported to enhance barrier function and attenuate intestinal inflammation, it remains unclear whether SLBZS exerts its therapeutic effects through the AhR/CYP1A1/NF-κB signaling cascade. Building upon this rationale, the present study is the first to hypothesize that SLBZS may mediate its pharmacological activity *via* the AhR/CYP1A1/NF-κB signaling axis. A comprehensive strategy combining network pharmacology, molecular docking, and *in vivo* experimentation will be employed to elucidate the mechanistic basis of SLBZS, spanning from target prediction to pathway-level validation across the entire therapeutic cascade.

In this study, we first identified the key molecular targets of SLBZS for UC treatment through network pharmacology analysis, and subsequently conducted animal experiments to validate its therapeutic effects, highlighting its antioxidant, anti-inflammatory, and intestinal barrier-repair properties in DSS-induced UC mice. Molecular docking and molecular experiments ultimately confirmed that SLBZS alleviates DSS-induced colitis symptoms in mice by regulating the AhR/CYP1A1/NF-κB signaling pathway [26, 27].

## 2 MATERIALS AND METHODS

### 2.1 Reagents

TCM ingredients were sourced from Yancheng Hospital of Traditional Chinese Medicine. The drug composition of SLBZS is shown in Table **1**. DSS with a 36-50 kDa MW (MP Biomedicals, California, USA); Mesalamine (Ethypharm Pharmaceutical, Shanghai, China); Antibodies specific to inflammasome components including CYP1A1, ZO-1, Occludin, Beta Tubulin, NF-κB (p65), p-NF-κB (p-p65), and IL-6 (Proteintech, Jiangsu, China); ELISA kits for IL-6, IL-18, IL-1β, MPO, and TNF-α (Beyotime Biotechnology, Jiangsu, China); Kits for MDA, GSH, and SOD (Nanjing Jiancheng Bioengineering Institute, Jiangsu, China).

### 2.2 SLBZS Preparation and UPLC-MS/MS Analysis

The SLBZS decoction was formulated according to classical proportions: Codonopsis pilosula (Franch.) Nannf (20 grams), Poria cocos (Schw.) Wolf (15 grams), Atractylodes macrocephala Koidz (20 grams), Dioscorea opposite Thunb. (20 grams), Dolichos Lablab L. (15 grams), Semen Nelumbinis. (10 grams), Glycyrrhiza uralensis Fisch. (10 grams), Amomum villosum Lour. (10 grams), Coix lacryma-jobi L.(10 grams), and Platycodon grandiflorus (10 grams). The herbal mixture was soaked in distilled water at a 1:10 (w/v) proportion to facilitate extraction. The extraction process involved two consecutive 30-minute reflux cycles using distilled water, after which the solution was concentrated under vacuum to yield a final volume of 62mL. For quantitative component analysis, 0.2 grams of SLBZS aqueous extract was precisely measured, dissolved in 10 mL of methanol, and sonicated for 30 minutes. The resulting solution was subsequently centrifuged at 12,000 rpm for 10 minutes. The clear supernatant was then passed through a 0.22 μm filter membrane, followed by dilution within the linear range of the calibration curve prior to analysis. The aqueous extract of SLBZS was then treated with methanol, and the resulting mixture was subjected to centrifugation to isolate the supernatant, which was subsequently analyzed using UPLC-MS/MS. Detailed experimental conditions, including instrument models, operating parameters, and analytical procedures, are provided in the Supplementary Materials **S1**.

The SLBZS solution and reference compound solutions were analyzed by UPLC using a hybrid Quadrupole-Exactive Orbitrap instrument with an ACQUITY UPLC BEH C18 column (2.1 × 100mm; 1.7μm) maintained at 40°C. The parameters included a flow rate of 0.5 mL/min and a 3 μL injection volume, with 0.1% formic acid in water (A) or acetonitrile (B) representing the mobile phase. The linear elution gradient was programmed as follows: from 0 to 11 minutes, solvent A decreased from 85% to 25%; between 11 and 12 minutes, it further dropped to 2%; an isocratic hold at 2% A was maintained from 12 to 14 minutes; at 14 to 14.1 minutes, the proportion of A rose back to 85%; and from 14.1 to 16 minutes, an isocratic phase at 85% A was applied. Mass Spectrometry (MS) and tandem MS (MS/MS) data were matched against the proprietary TCM metabolite database (MJBIOTCM; Shanghai Majorbio Bio-Pharm Technology Co., Ltd.). The MS parameters were sheath and auxiliary gas flow rates of 50 and 13 Arb, respectively, with temperatures of 320°C and 350°C for the ion transfer tube and vaporizer, respectively, a full MS resolution of 70,000 and MS/MS resolution of 17,500, collision energies of 20/40/60 in Normalized Collision Energy (NCE) mode; and spray voltages of 3.5 kV (positive mode) or 3 kV (negative mode). Furthermore, reference solutions of sinensetin, Kaempferol, formononetin, and luteolin were prepared across a concentration range of 1-500 ng/mL to construct calibration plots (R^2^ > 0.996). The levels of these constituents in SLBZS (expressed in μg/g) were determined *via* the external standard approach, with each sample analyzed in triplicate.

### 2.3 Animal Experiment

C57BL/6 male mice (*n* = 36, 6–8 weeks old, 20 ± 2 grams) were purchased from Jiangsu Huachuang Xinnuo Pharmaceutical Technology Co., Ltd. Animals were kept under controlled environmental conditions with free access to standard chow and water. After a one-week acclimatization period, the mice were randomly divided into six groups (*n* = 6): Control, DSS, DSS + L-SLBZS, DSS + M-SLBZS, DSS + H-SLBZS, and mesalazine groups. Group sizes were determined by power analysis (effect size f = 0.5, α = 0.05) to achieve > 80% power for a one-way ANOVA, following the 3Rs principle.

Throughout the study, the control group received regular drinking water, while DSS-exposed groups were given a 2.5% DSS solution from day 1 to day 10. SLBZS or mesalazine was administered *via* oral gavage once daily at 10:00 a.m. from day 4 to day 10 at doses of 3.5, 7, and 14 g/kg for the low, medium, and high SLBZS groups, respectively. The control group received distilled water. Body weight, stool consistency, and presence of occult or visible blood were recorded daily to calculate the Disease Activity Index (DAI) according to predefined criteria (Supplementary Table **1**).

All experimental procedures were performed following the Guidelines for the Care and Use of Laboratory Animals and were approved by the Animal Ethics Committee of Jinling Hospital, Nanjing University of Chinese Medicine (Approval No. 2023JLHGZRDWLS-00087).

### 2.4 Network Pharmacology Analysis

The SLBZS components identified by UPLC-MS/MS were linked to protein targets using the TCMSP database (https://old.tcmsp-e.com/tcmsp.php) [28, 29] and standardized *via* the UniProt database (https://www.uniprot.org/). UC-associated targets were retrieved from the GeneCards database (https://www.genecards.org/), focusing on those with relevance scores above the median quartile. The Venny 2.1 tool was utilized to generate Venn diagrams for SLBZS and UC (https://bioinfogp.cnb.csic.es/tools/venny/), thereby enabling the identification of overlapping targets. A Protein-Protein Interaction (PPI) network was constructed using Cytoscape (https://cn.string-db.org/), followed by the use of the online platform DAVID for Gene Ontology (GO) annotation of shared targets. Based on the Metascape database (https://metascape.org/gp/index.html), we further performed Kyoto Encyclopedia of Genes and Genomes (KEGG) pathway enrichment analysis, with the results visualized through the Micro-Biotech platform. (Full details, including database versions, screening parameters, and statistical thresholds, are provided in the Supplementary Materials **S2**).

### 2.5 ELISA

Whole blood sampling was performed in the ocular region of the mice, followed by 20 minutes of centrifugation at 4,000 rpm to extract serum. Using the ELISA kits, pro-inflammatory cytokine levels were determined, and concentrations of oxidative stress indicators were assessed.

### 2.6 Molecular Docking

Molecular docking is a crucial technique for examining the interactions between ligands and their corresponding targets. The present study employed the AutoDock Vina docking tool to analyze the interaction of small molecular compounds with protein structures. Sinensetin, Kaempferol, formononetin, and luteolin were obtained from the PubChem (https://pubchem.ncbi.nlm.nih.gov) repository and employed as representative small-molecule ligands. Concurrently, the three-dimensional structures of CYP1A1, AHR, and NF-κB1 were sourced from the Protein Data Bank (PDB) and designated as target receptors (https://www.rcsb.org). Given the analysis results, we set the cutoff of ligand-receptor binding affinity to -5.0 kcal/mol [30, 31], below which the ligand-receptor binding is considered stable. The results were visualized using PyMOL software, facilitating interpretation and enabling thorough analysis. Detailed docking procedures, including protein preparation, grid box parameters, and software versions, are provided in the Supplementary Materials **S3**.

### 2.7 Immunofluorescence

During the animal experiments, colonic tissue samples of the mice were collected and then thoroughly washed using physiological saline, followed by paraffin embedding for the purpose of Immunofluorescence (IF) staining. Subsequently, antigen retrieval was carried out, and then a blocking step with 3% Bovine Serum Albumin (BSA) was implemented, followed by incubating tissue sections overnight with an anti-ZO-1and Occludin antibody at 4°C. Next, the sections were treated for 1 hour using Cy3-conjugated donkey anti-rabbit IgG at room temperature, followed by staining the cell nuclei with DAPI.

### 2.8 Western Blotting (WB)

Colonic tissue underwent preparation, followed by the addition of RIPA lysis buffer. The mixture was agitated and centrifuged to separate the supernatant. Next, Proteins were separated by SDS-PAGE and then transferred onto a PVDF membrane. The membrane was blocked with 5% skim milk for 2 hours, followed by overnight incubation with the primary antibody. The following day, the membrane was treated for an additional 2 hours using the secondary antibody, and protein expression was evaluated *via* chemiluminescence detection.

### 2.9 H&E Staining

Tissues were immersed in 4% neutral formaldehyde for three weeks, followed by paraffin embedding and sectioning (4 μm). Staining with H&E followed standard protocols. A pathologist, unaware of the experimental conditions, conducted histological analysis, using imaging under light microscopy (Nikon, Japan).

### 2.10 Statistical Analyses

Experimental data were statistically analyzed using GraphPad Prism and SPSS 24.0. One-way Analysis of Variance (ANOVA) was performed, followed by the post-hoc Student-Newman-Keuls (SNK) test, with statistical significance set at *P* < 0.05.

## 3 RESULTS

### 3.1 1UPLC-MS/MS-based Identification of SLBZS Components

The corresponding ion chromatograms of SLBZS components identified using UPLC-MS/MS are depicted in Fig. (**1A**-**B**). A total of 458 unique components were identified within SLBZS (Supplementary Table **2**), with the relative abundance of each compound in the SLBZS illustrated in Fig. (**1C**). The primary active components identified comprise flavonoids, terpenes, coumarins, and their derivatives. According to the alignment results with a self-compiled traditional Chinese medicine metabolite library and assessment of signal intensity, four principal constituents with comparatively high levels, clearly defined molecular structures,and prospective bioactivity were identified and highlighted in Fig. (**1D**-**G**): sinensetin, kaempferol, formononetin, and luteolin. These flavonoid-based molecules also exhibited notable target-binding affinity in both network pharmacological evaluation and molecular docking simulations, thus being selected as focal candidates for further mechanistic exploration.

### 3.2 SLBZS Exerts a Mitigating Effect on Experimental Colitis Induced by DSS

**Fig. (2):**
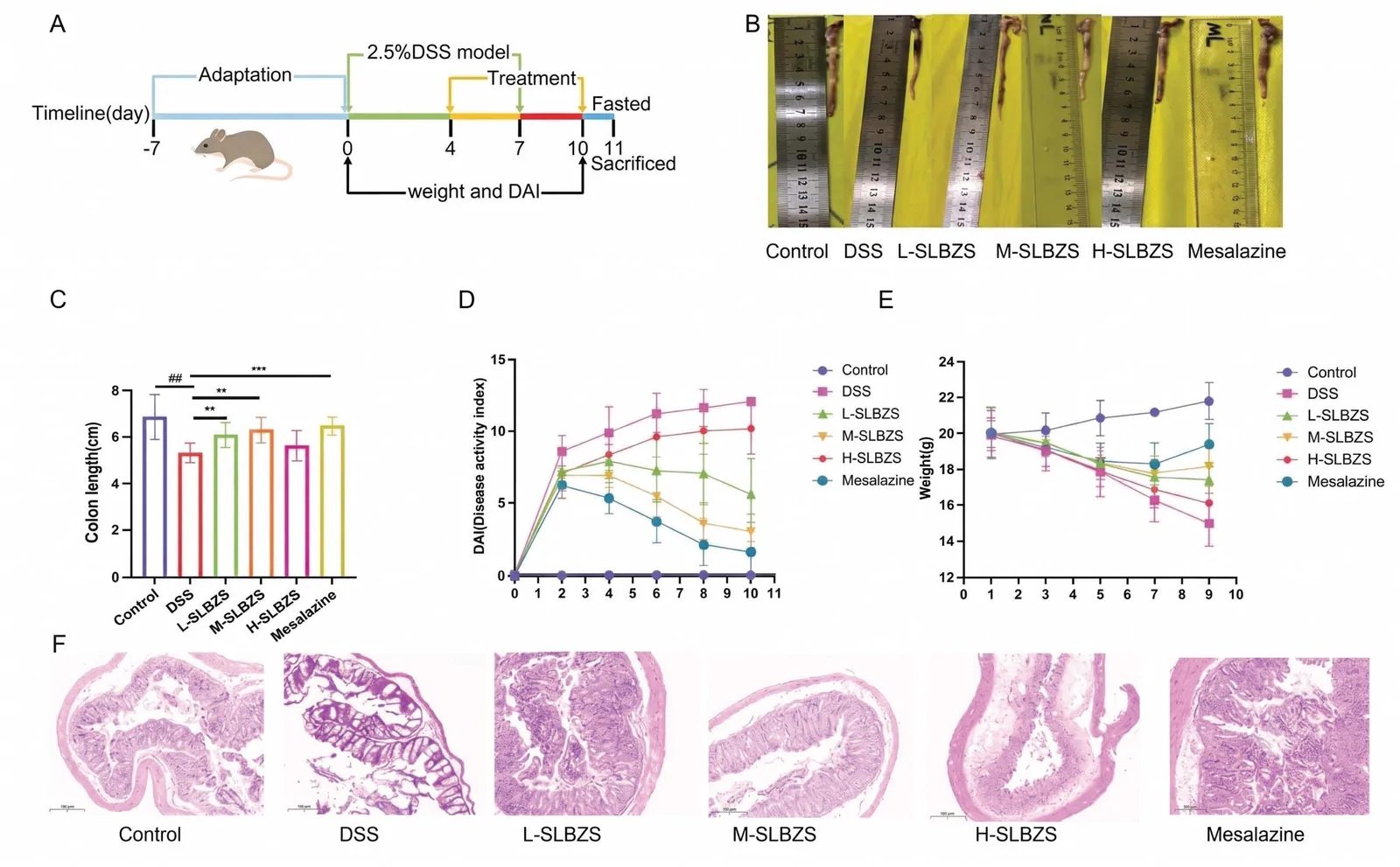
SLBZS is Effective in Treating UC Mice. (**A**) Establishment of DSS-colitis mice model and drug treatment. (**B-C**) Colon length. (**D**) DAI scores. (**E**) Body weight changes in mice. (**F**) Representative H&E staining images of the distal colon from mice. Scale bar represents 200μm. ^##^*P* < 0.05 *vs.* control group, **P < 0.01, ***P < 0.001 *vs.* DSS group.

Fig. (**2A**) depicts the experimental procedure. A standard weight gain was achieved in the control group on a regular diet; however, following 5 days of treatment with a 2.5% DSS aqueous solution, a significant weight loss was noted, along with hematochezia and general malaise, confirming that the UC model was successfully induced (*P* < 0.05). After SLBZS was administered, a marked improvement in symptoms was noted, especially in L-SLBZS, M-SLBZS, and mesalamine groups (Fig. (**2B**-**C**). Notable improvements in body weight and DAI scores were observed following treatment with L-SLBZS, M-SLBZS, and mesalamine groups (Fig. (**2D**-**E**) (*P* < 0.01, *P* < 0.001). Histopathological analysis revealed that the control group exhibited normal colonic architecture, whereas significant damage occurred to the intestinal structure of the DSS group, marked by extensive infiltration of inflammatory cells. However, we observed a marked reduction in pathological changes, inflammatory cell infiltration, and intestinal tissue damage in groups treated with SLBZS and mesalamine, particularly the mesalamine, L-SLBZS, and M-SLBZS groups (Fig. **2F**) (*P* < 0.01, *P* < 0.001).

### 3.3 SLBZS Diminishes Inflammation and Elevates Antioxidant Levels

**Fig. (3):**
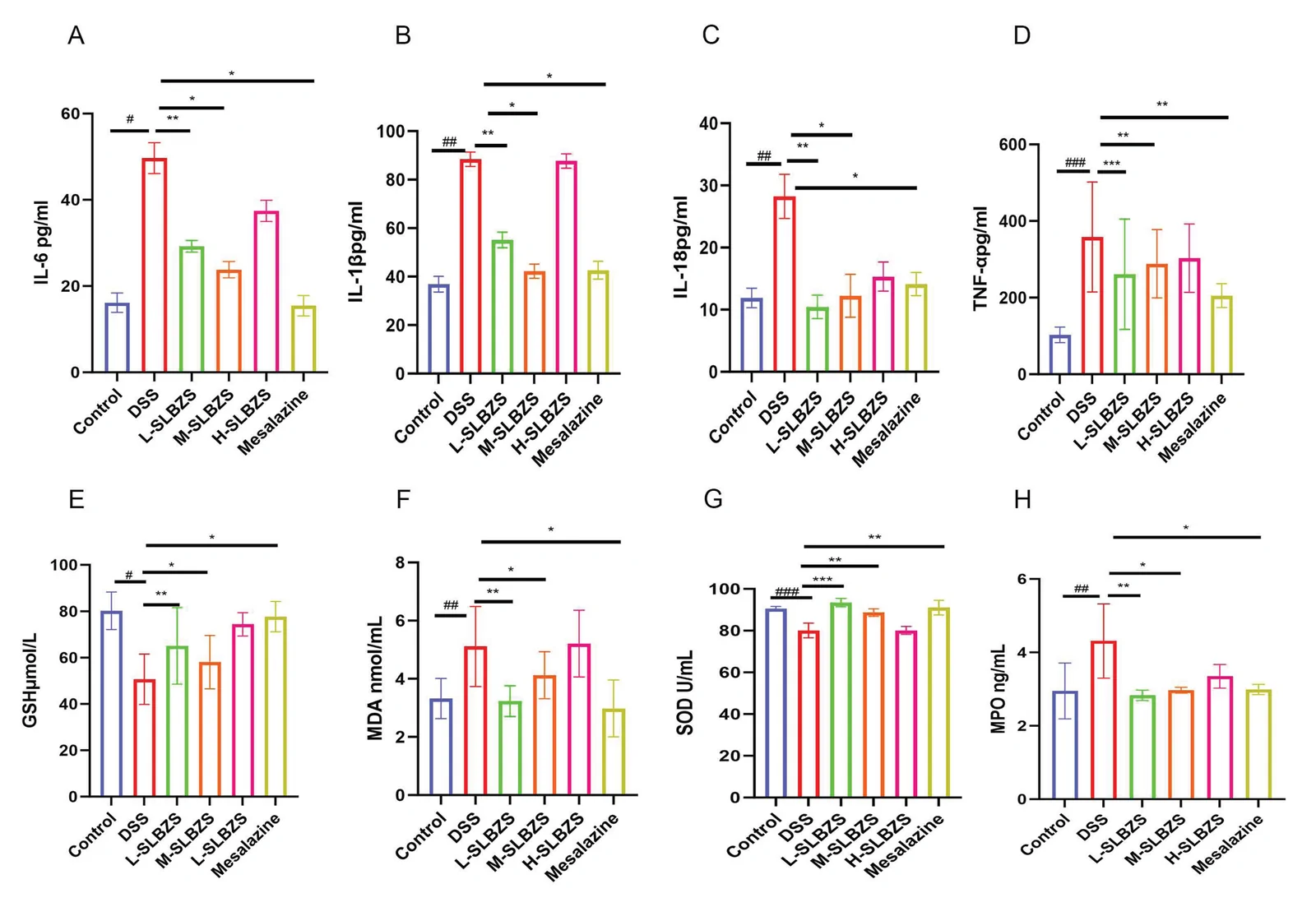
SLBZS diminishes inflammation and elevates antioxidant levels. (**A-D**) Determination of IL-6, IL-1β, IL-18, and TNF-α in serum. (**E-H**) Determination of MDA, SOD, GSH, and MPO in serum. ^#^*P* < 0.01, ^##^*P* < 0.001, ^###^*P* < 0.0001 *vs*. control group, *P < 0.05, **P < 0.01, ***P < 0.001 *vs.* DSS group.

To examine the therapeutic effect mechanisms of SLBZS on UC, this study assessed inflammatory biomarkers in the serum of murine models and evaluated oxidative stress indicators in colonic tissues. The DSS group demonstrated markedly higher expression of IL-6, IL-18, IL-1β, and TNF-α than the control group (*P* < 0.01, *P* < 0.001, *P* < 0.001). Conversely, treatment with mesalamine and L-SLBZS caused a considerable reduction in their expression (Fig. **3A**-**D**) (*P* < 0.05, *P* < 0.01). Additionally, the DSS group exhibited higher Malondialdehyde (MDA) and Myeloperoxidase (MPO) levels, as well as lower Glutathione (GSH) and Superoxide Dismutase (SOD) concentrations than the control group (*P* < 0.01, *P* < 0.001, *P* < 0.001). Treatment with mesalamine and L-SLBZS effectively mitigated these oxidative stress markers, resulting in reduced MDA and MPO levels, while enhancing GSH and SOD levels (Fig. **3E**-**H**) (*P* < 0.05, *P* < 0.01). These findings indicate that SLBZS could alleviate the pathogenesis of UC by modulating inflammatory responses and reducing oxidative stress.

### 3.4 SLBZS Could Repair the Intestinal Barrier

**Fig. (4):**
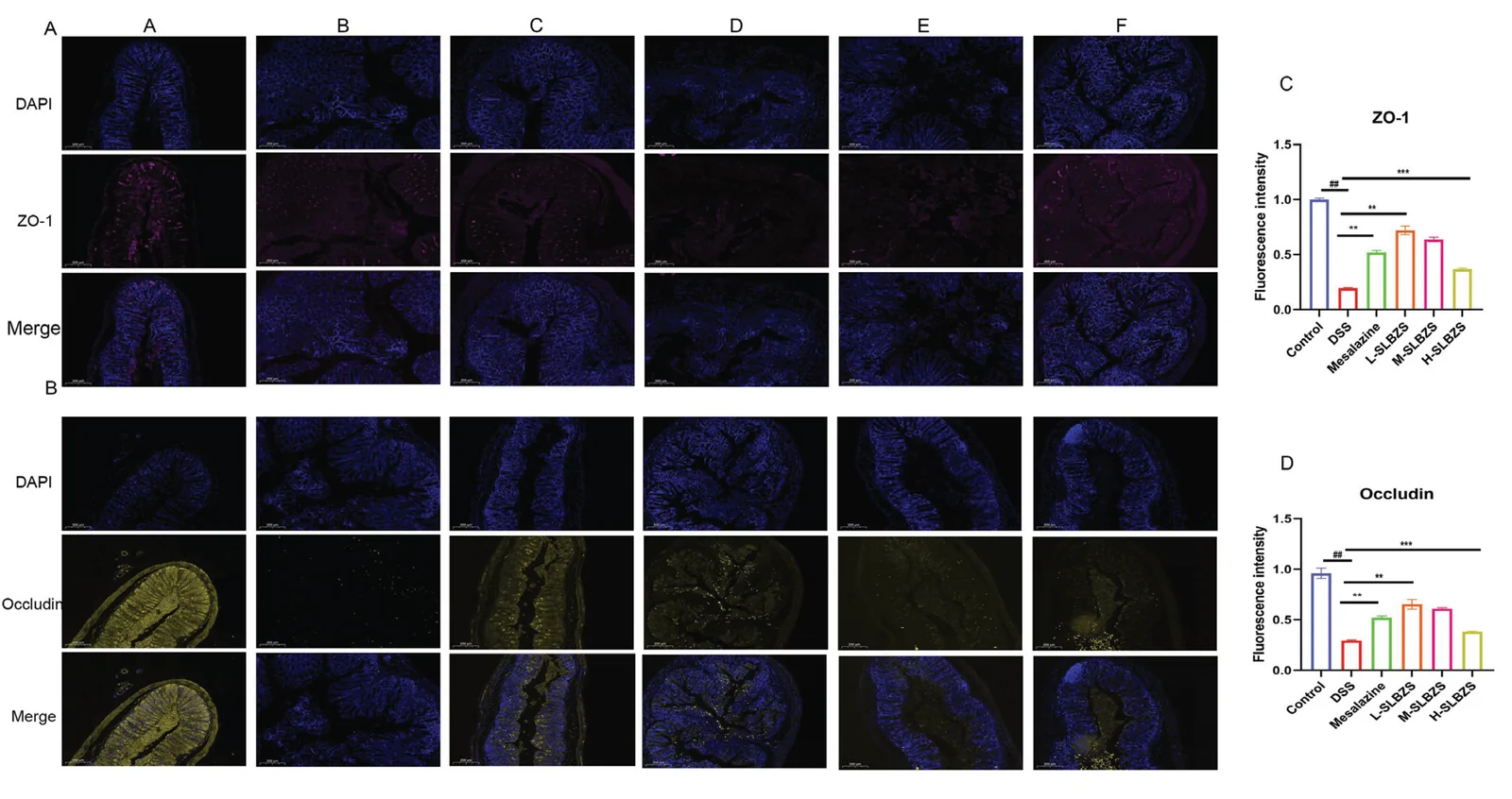
SLBZS Demonstrates Therapeutic Efficacy in a UC Mouse Model. (**A**) ZO-1 expression in the colon was assessed by immunofluorescence. (**B**) Occludin expression in the colon was assessed by immunofluorescence. (**C-D**) Relative density of ZO-1 and Occludin positive regions in immunofluorescence. A: control group, B: DSS group, C: mesalazine group, D: DSS + L-SLBZS group, E: DSS + M-SLBZS group, F: DSS + H-SLBZS group. ^##^*P* < 0.01 *vs.* control group, **P < 0.05, ***P < 0.01 *vs.* DSS group.

Immunofluorescence analysis revealed that the administration of SLBZS significantly ameliorated the impaired intestinal barrier function in colitis-induced mice. Quantitative assessment demonstrated a substantial reduction in the expression levels of the tight junction proteins Occludin and ZO-1 in the DSS-treated group compared to the controls (*P* < 0.01). Notably, both L-SLBZS and M-SLBZS treatments effectively upregulated Occludin expression and enhanced ZO-1 protein levels, as illustrated in (Fig. **4A**-**D**) (*P* < 0.05, *P* < 0.001).

### 3.5 Network Pharmacology Analysis

**Fig. (5):**
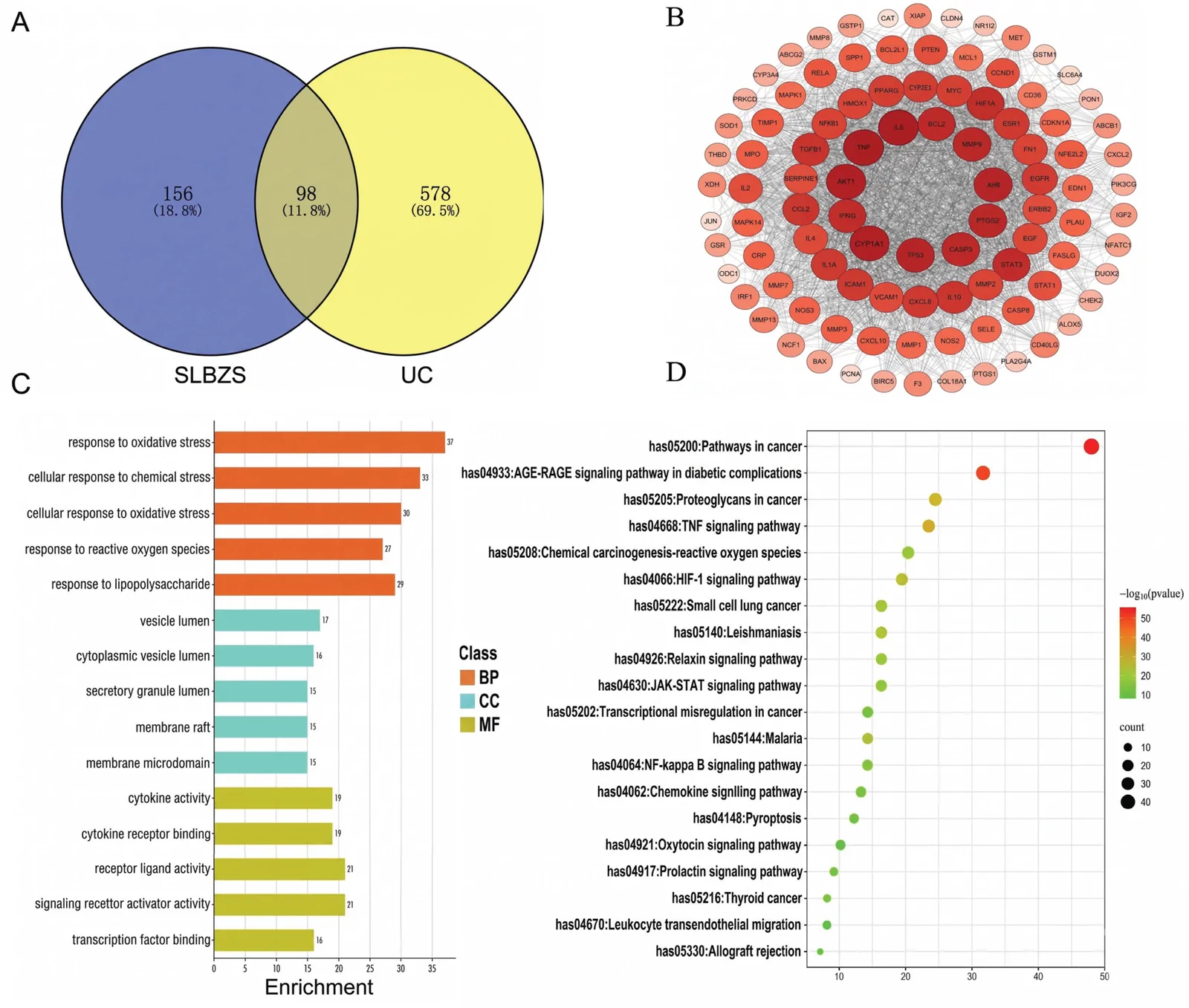
Network pharmacology analysis of SLBZS for colitis treatment. (**A**) Common targets between SLBZS and UC disease-related targets. (**B**) The PPI network of crucial genes. (**C**) GO functional enrichment analysis. (**D**) KEGG pathway enrichment analysis.

The molecular mechanisms through which SLBZS provides therapeutic benefits for UC were investigated using network pharmacology. Potential targets influenced by compounds identified through UPLC/MS-MS were screened, yielding 254 candidates using the TCMSP database. Subsequently, the targets associated with UC were retrieved from the GeneCards database, resulting in the identification of 676 targets (Supplementary Table **3**), with a relevance score greater than 4. A Venn diagram analysis was conducted for identifying the shared targets of SLBZS and UC, revealing 98 overlapping targets (Fig. **5A**, Supplementary Table **4**). A Protein-Protein Interaction (PPI) network constructed from these 98 DEGs highlighted NF-κB, AhR, CYP1A1, TNF, and NLRP3 as central hubs (Fig. **5B**). As revealed by the GO enrichment analysis, SLBZS primarily functions in the responses to oxidative stress, cellular reactions to chemical stress, vesicle lumen activities, cytokine activity, and the binding of cytokine receptors (Fig. **5C**). Moreover, KEGG pathway enrichment analysis revealed that SLBZS participates in various pathways, including the Nuclear Factor kappa-light-chain-enhancer of activated B cells (NF-κB) signaling, Tumor Necrosis Factor (TNF) signaling, pyroptosis signaling pathways, cancer-associated pathways, as well as the diabetic complication-associated, Advanced Glycation End Products-Receptor for Advanced Glycation End Products (AGE-RAGE) signaling pathway. These results highlight that the efficacy of SLBZS in treating UC is fundamentally associated with its modulatory effects on the intestinal barrier integrity, inflammatory processes, and oxidative stress responses (Fig. **5D**).

### 3.6 SLBZS Regulates the AHR/CYP1A1/NF-κB Axis

**Fig. (6):**
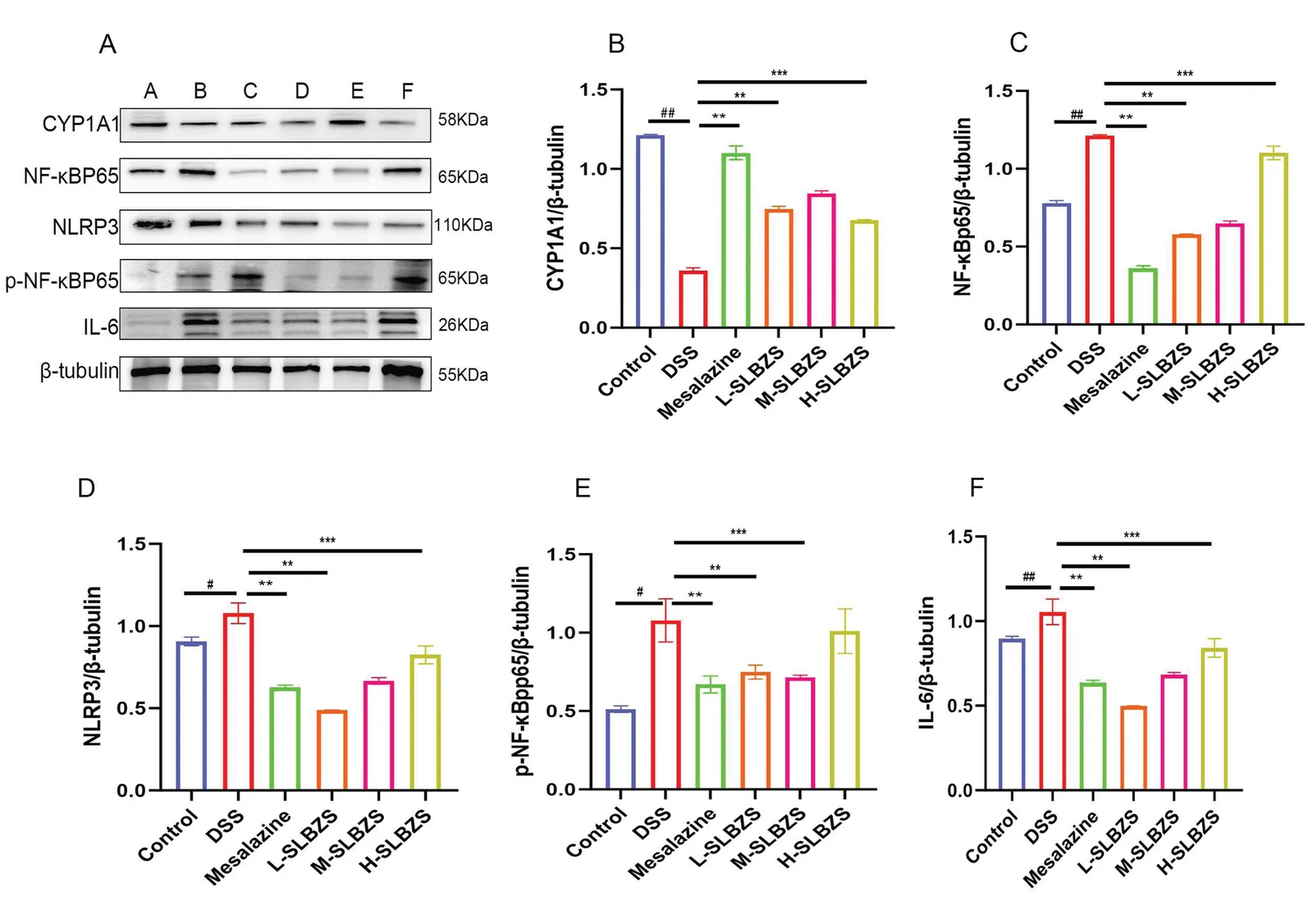
SLBZS alleviates colitis in mice through the AHR/CYP1A1/NF-κB axis. (**A-F**) Western blot analysis and semi-quantification of the relative levels in each group. A: control group, B: DSS group, C: mesalazine group, D: DSS + L-SLBZS group, E: DSS + M-SLBZS group, F: DSS + H-SLBZS group. ^#^*P* < 0.01, ^##^*P* < 0.001 *vs.* control group, **P < 0.05, ***P < 0.01 *vs.* DSS group.

As shown by network pharmacology, SLBZS enhanced the intestinal barrier and mitigated colitis by modulating the AhR/CYP1A1/NF-κB signaling pathway. The levels of CYP1A1, NF-κB, and downstream components in this pathway were examined by WB. The DSS group had considerably lower CYP1A1 levels and significantly higher NF-κB (p65), IL-6, NLRP3, and p-NF-κB (p-p65) concentrations than the control group (*P* < 0.01). After SLBZS or mesalazine treatment, NF-κB (p65), IL-6, NLRP3, and p-NF-κB (p-p65) levels decreased significantly, while CYP1A1 levels increased (Fig. **6A**-**F**) (*P* < 0.05).

### 3.7 Molecular Docking Analysis

**Fig. (7):**
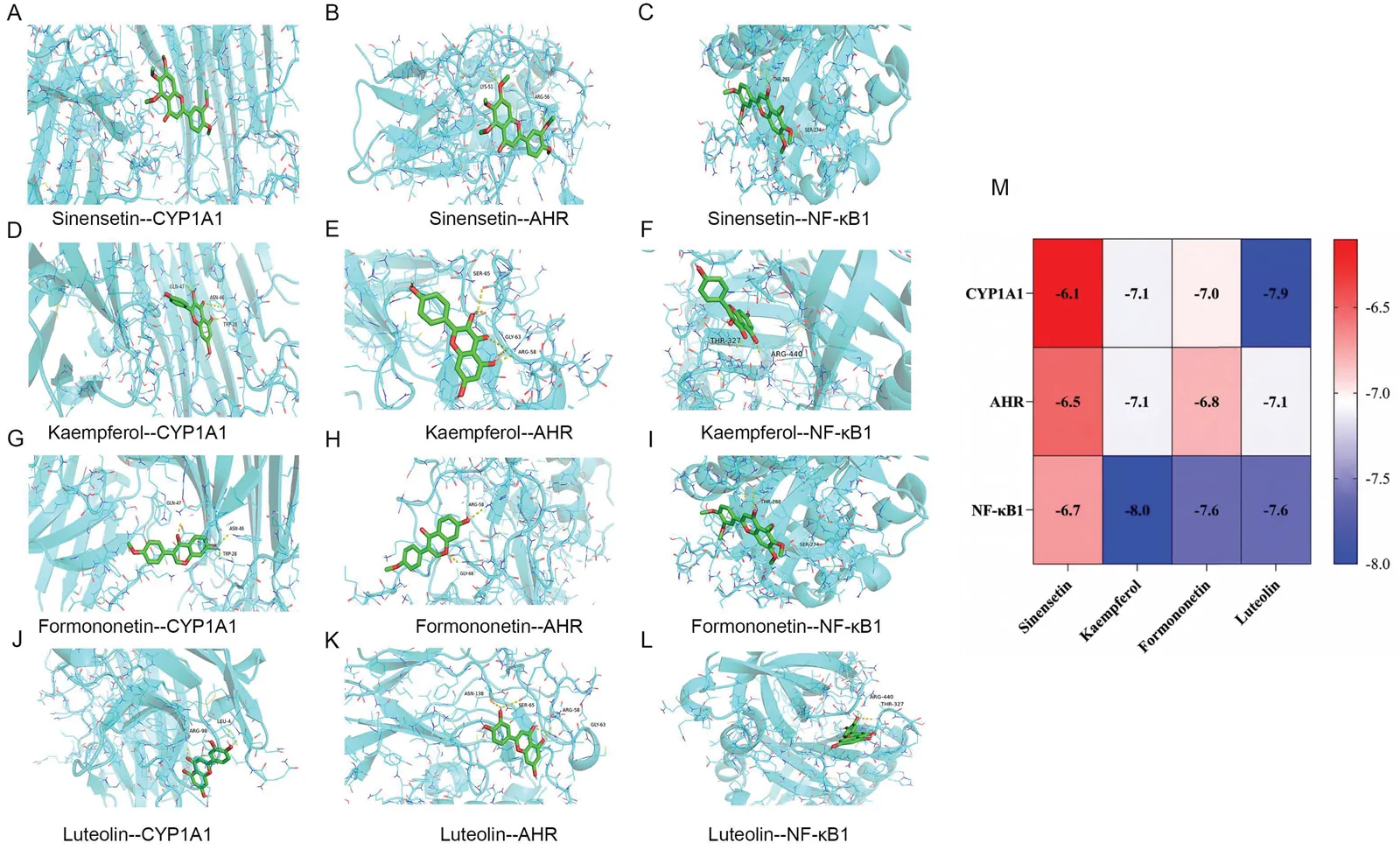
Molecular docking analysis. (**A-L**) Molecular docking analysis of AHR, CYP1A1, and NF-Κb1 proteins with Sinensetin, Kaempferol, Formononetin, and Luteolin. (**M**) Heat map.

A ligand-receptor binding energy under -5 kcal/mol indicates that docking is feasible, with lower binding energies signifying more stable interactions. The four primary components identified *via* MS were individually docked with CYP1A1, AhR, and NF-κB1 (Fig. **7A**-**L**), and all demonstrated binding energies below -5 kcal/mol (Fig. **7M**). This observation signifies a stable interaction between the ligands and their corresponding receptors.

## 4 DISCUSSION

Ulcerative Colitis (UC), a persistent inflammatory disorder of the colonic mucosa, manifests as a relapsing-remitting disease pattern with an escalating incidence among adolescents and young adults [32]. This condition induces multidimensional health deterioration encompassing physical discomfort, psychological distress, and socioeconomic impairment. Emerging evidence [33, 34] underscores the therapeutic potential of phytotherapeutic interventions, particularly Traditional Chinese Medicine (TCM) formulations, in achieving endoscopic remission and restoring immune homeostasis in UC management. SLBZS has been utilized in clinical settings for the management of abdominal pain and diarrhea. Recent pharmacological investigations have demonstrated that SLBZS alleviates symptoms associated with colitis and reduces inflammatory responses [35].

The analysis of chemical composition is of vital significance in understanding the pharmacological attributes and mechanisms of action related to Traditional Chinese Medicine (TCM) herbs [36-38]. In this research, the chemical profiling of SLBZS was carried out using Ultra-Performance Liquid Chromatography in conjunction with tandem Mass Spectrometry (UPLC-MS/MS). The findings indicated that the predominant classes of compounds identified within SLBZS included flavonoids, terpenes, and coumarins. Comparison of the detected compounds against an in-house TCM metabolite database, combined with an assessment of signal intensity, enabled the identification of four principal constituents-sinensetin, kaempferol, formononetin, and luteolin-characterized by high abundance, defined molecular structures, and putative biological activity.

Previous studies have explored the functional roles of these compounds using Lipopolysaccharide (LPS)-or Dextran Sulfate Sodium (DSS)-induced inflammatory models. Sinensetin (SIN) [39], a type of polymethoxyflavone, was shown to significantly suppress the transcription of multiple pro-inflammatory mediators, including IL-1α, IL-1β, IL-6, NLRP3, STAT3, and Bcl2l1, while simultaneously enhancing the mRNA levels of anti-inflammatory cytokines, such as IL-4, IL-10, and IL-12α. These actions were strongly associated with the prevention of NF-κB p65 translocation into the nucleus [40]. In intestinal epithelial/endothelial co-culture models challenged with LPS, Laempferol (Kae) [41] lowered IL-8 release from Caco-2 monolayers and restored epithelial barrier integrity, as evidenced by the recovery of Transepithelial Electrical Resistance (TEER), primarily through modulation of NF-κB signaling [42, 43]. Formononetin (FN) [44], an isoflavone compound, has been recognized as an important modulator of macrophage phenotype in DSS-induced colitis, facilitating microbiota-dependent regulation of the M1/M2 balance and mitigating colitis progression *via* reprogramming of immune metabolism [45, 46]. Luteolin (Lut) [47] demonstrated a dual mode of action by reshaping intestinal microbial composition and suppressing inflammatory metabolic pathways, thereby promoting barrier repair and ameliorating the inflamed mucosal environment [48, 49].

Importantly, these four major compounds are flavonoids [50, 51] or isoflavonoids [52, 53] of natural origin, which have been documented as potential endogenous ligands for the Aryl hydrocarbon Receptor (AhR). Interaction with AhR can trigger the AhR-CYP1A1 signaling cascade, leading to the attenuation of NF-κB-driven inflammatory pathways, the upregulation of tight junction-associated proteins, restoration of epithelial barrier integrity, and a reduction in oxidative damage. Such pleiotropic actions are presumed to involve, at least partially, AhR-dependent regulatory processes, offering strong molecular support for the concept that SLBZS mediates its beneficial effects through modulation of the AhR/CYP1A1/NF-κB signaling network.

To assess the therapeutic efficacy of SLBZS, a murine model of colitis was induced using 2.5% DSS [54, 55], followed by treatment with SLBZS. Our findings indicated that SLBZS effectively mitigated weight loss, improved the Disease Activity Index (DAI), and led to a reduction in colonic length. Furthermore, SLBZS was shown to alleviate the inflammatory conditions of the colon, as evidenced by a decrease in the expression of pro-inflammatory cytokines, specifically IL-6, IL-18, and IL-1β. The gut barrier serves as the primary defense mechanism against external pathogens, including bacteria and viruses [56]. Our findings indicate that SLBZS effectively repairs damaged colonic tissues and reinstates TJ integrity [57]. SLBZS significantly upregulated colonic TJ proteins occludin and zo-1, as demonstrated by immunofluorescence analysis. UC pathogenesis has been reported to be closely associated with oxidative stress [58-60]. Our experiments utilizing animal models revealed a reduction in MDA and MPO levels, alongside an enhancement in GSH and SOD expression after SLBZS treatment. This observation suggests that SLBZS may exert protective effects on intestinal epithelial cells against free radical-induced damage by mitigating oxidative stress and restoring antioxidant enzyme activity.

To explore the mechanisms by which SLBZS modulates inflammation and restores the intestinal barrier, a network pharmacological analysis was conducted based on shared targets between UC and SLBZS components identified *via* UPLC-MS/MS. Key targets included NF-κB, AhR, CYP1A1, TNF, and NLRP3. Subsequent GO and KEGG pathway enrichment analyses suggested that SLBZS exerts its therapeutic effects on UC through various biological processes, such as the response to oxidative stress and cellular chemical stress, as well as pyroptosis and cancer-related signaling pathways. Notably, the significant relationships established with the NF-κB signaling pathway and the AGE-RAGE signaling pathway offer profound molecular understanding of the mechanisms underlying the beneficial effects of SLBZS in UC treatment.

CYP1A1, a crucial enzyme within the cytochrome P450 superfamily, is instrumental in metabolizing exogenous compounds, including polycyclic aromatic hydrocarbons, pharmaceuticals, and environmental toxins. It modulates the inflammatory balance through two distinct mechanisms: first, it catalyzes the epoxidation of Arachidonic Acid (ARA), generating anti-inflammatory Epoxy Fatty Acids (EpFAs) and Epoxyeicosatrienoic acids (EETs) [61]; second, it facilitates hydroxylation, producing pro-inflammatory Hydroxyeicosatetraenoic acids (HETEs). In individuals with inflammatory intestinal disorders [62], CYP1A1 expression declines, leading to increased oxidative stress, disrupted intestinal barrier integrity, and an elevated release of pro-inflammatory mediators [63, 64].

The expression of this enzyme is tightly controlled by the AhR, a ligand-dependent transcription factor that is extensively present in epithelial and immune cells [65]. AhR regulates critical cellular processes, such as proliferation, apoptosis, differentiation, and immune responses. Upon interacting with endogenous ligands, plant-derived molecules, or environmental toxins, AhR [66, 67] dimerizes with ARNT and migrates to the nucleus, where it binds to enhancer regions of target genes, including CYP1A1, to initiate transcription. Research indicates that in UC patients, AhR expression and activity are significantly diminished, resulting in reduced protective metabolites and compromised barrier function. Activation of AhR using agonists like FICZ and quercetin has been shown to alleviate intestinal inflammation *via* two primary mechanisms [68]: enhancing the expression of tight junction proteins and suppressing myosin light chain kinase phosphorylation, thereby improving epithelial barrier function.

NF-κB, a crucial transcription factor, plays a central role in modulating the NLRP3 inflammasome and exacerbating colitis through various pathways [69, 70]. In Inflammatory Bowel Disease (IBD), aberrant NF-κB signaling, activated through pathways such as TLR4/MyD88 or TNF-α receptors, binds directly to the promoter region of NLRP3 [71, 72], markedly upregulating transcription of inflammatory molecules, including NLRP3, pro-IL-1β, and pro-IL-18. This cascade drives excessive production of inflammatory cytokines (*e.g.*, IL-1β, IL-6, TNF-α) and growth factors (*e.g.*, VEGF, FGF) [73, 74], while also triggering caspase-1 activation *via* the NLRP3 inflammasome. This leads to Gasdermin D-mediated pyroptosis, perpetuating a cycle of chronic inflammation [75].

**Fig. (8):**
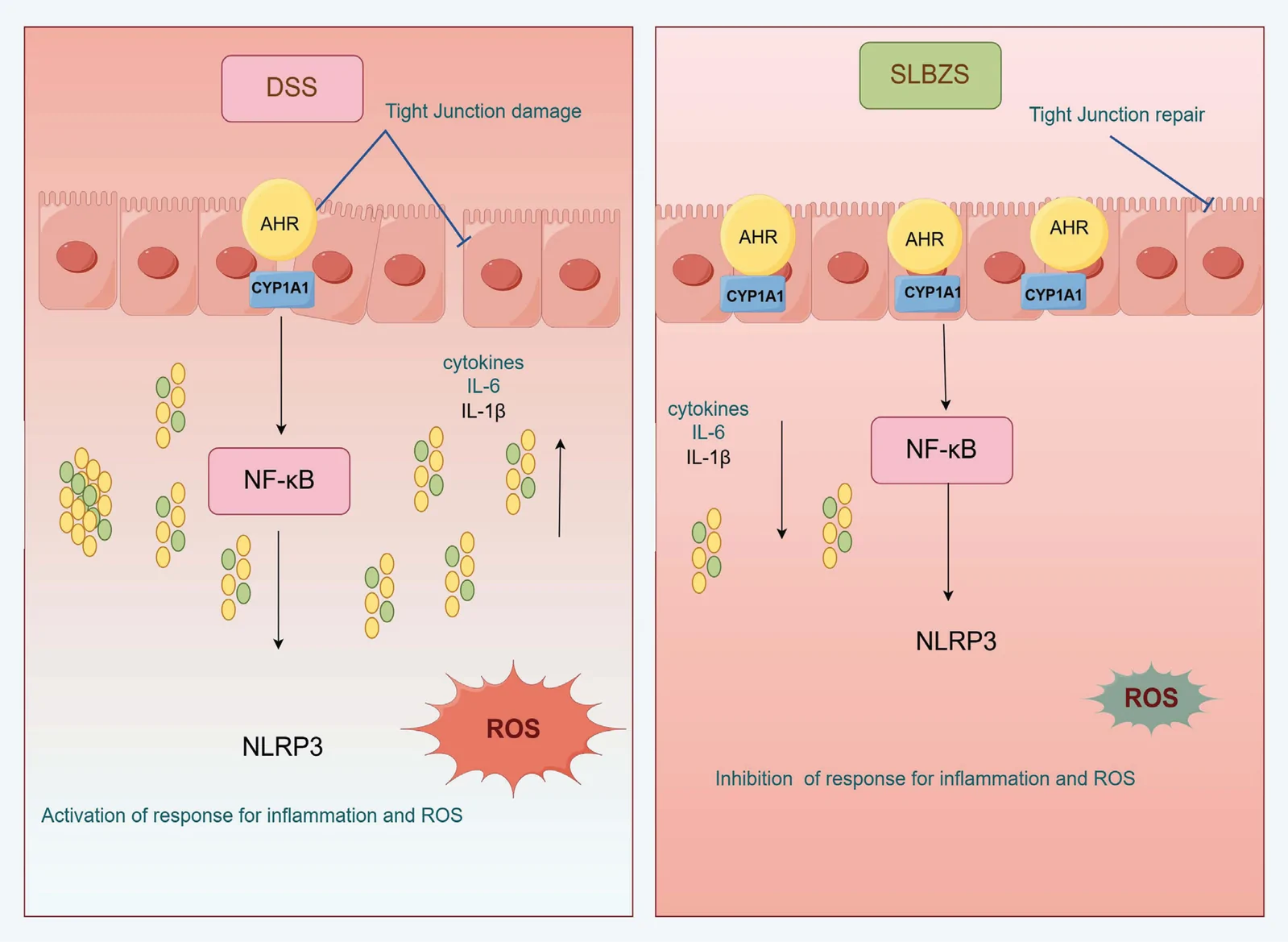
Schematic diagram depicting the mechanism of SLBZS in colitis treatment.

The AhR/CYP1A1/NF-κB axis is critically involved in regulating both physiological and pathological processes. Activation of AhR has been shown to counteract NF-κB signaling, mitigating inflammatory responses. Conversely, CYP1A1 metabolites may intensify UC-related inflammation through activation of the NF-κB pathway. Moreover, NF-κB can downregulate AhR expression or function while promoting the release of key inflammatory cytokines, including TNF-α, IL-1β, and IL-6, which contribute to the pathogenesis of UC. A Protein-Protein Interaction (PPI) network analysis has identified CYP1A1 and NF-κB as crucial therapeutic targets for colitis treatment, particularly in the context of SLBZS-based interventions. Additionally, molecular docking analyses confirm that the primary constituents of SLBZS-namely Sinensetin, Kaempferol, Formononetin, and Luteolin-exhibit significant binding stability with AhR, CYP1A1, and NF-κB, implying that these compounds may exert their therapeutic effects *via* direct interaction with these target proteins. This investigation validates the safety and efficacy of TCM formulations in treating colitis, laying a scientific foundation to support the clinical utilization of SLBZS in colitis management. These findings highlight SLBZS as a promising modulator of the AhR-CYP1A1-NF-κB axis in UC treatment (Fig. **8**). Future research should utilize genetic modification strategies, such as CYP1A1 knockout models, to confirm target specificity. Additionally, a thorough phytochemical profiling of SLBZS is necessary to identify its bioactive compounds, which will aid in the clinical development of this traditional medicinal formulation.

From a broader clinical standpoint, the present results underscore the potential of multi-constituent TCM preparations, such as SLBZS, to serve as valuable complements to existing UC treatment regimens. In contrast to conventional single-target therapeutics, SLBZS demonstrates multi-level and multi-pathway modulation, encompassing the regulation of inflammatory processes, oxidative stress responses, epithelial barrier integrity, and gut microbiota balance. This broad-spectrum activity aligns with emerging paradigms in precision medicine and combination therapy. In comparison to biologics or JAK inhibitors, which typically act on specific immune signaling routes and may carry immunosuppression-related risks, SLBZS displays a more comprehensive immunoregulatory capacity and a favorable safety profile, supporting its feasibility for prolonged administration. Moreover, the identification of AhR and CYP1A1 as pivotal molecular nodes provides a mechanistic bridge between phytotherapy and next-generation drug discovery strategies aimed at fine-tuning host-microbe and immune-epithelial crosstalk, thereby indicating its promise as an adjunct to biologics, targeted small molecules, or microbiota-centered interventions.

## CONCLUSION

This study examines the underlying mechanisms by which the traditional Chinese medicine SLBZS contributes to the treatment of UC. Evidence from our experiments suggests that SLBZS acts through a combination of molecular targets and biological pathways, most prominently by promoting activation of the AhR-CYP1A1 signaling route while restraining inflammatory activity mediated by NF-κB. These synergistic actions contribute to the repair of intestinal tight junctions, alleviate oxidative damage, and reduce abnormal immune responses in mice with DSS-induced colitis. By revealing these mechanistic associations, the research connects long-standing empirical applications with contemporary pharmacological interpretation. Furthermore, the identification of AhR and CYP1A1 as pivotal modulators suggests new points of intervention for ulcerative colitis and other diseases involving compromised epithelial barriers. From a clinical translation perspective, the findings justify the incorporation of SLBZS into therapeutic regimens, whether used independently for less severe disease or combined with standard medical treatments, thereby broadening safe and effective management options. Although these results are consistent with selected previous reports and support the reliability of our methodology, the proposed mechanisms should be further validated through complementary *in vitro* and *in vivo* studies. In addition, the phytochemical analysis focused on major constituents, leaving the potential contributions of minor components to be clarified in future research to deepen the understanding of SLBZS’s active material basis.

## Figures and Tables

**Fig. (1):**
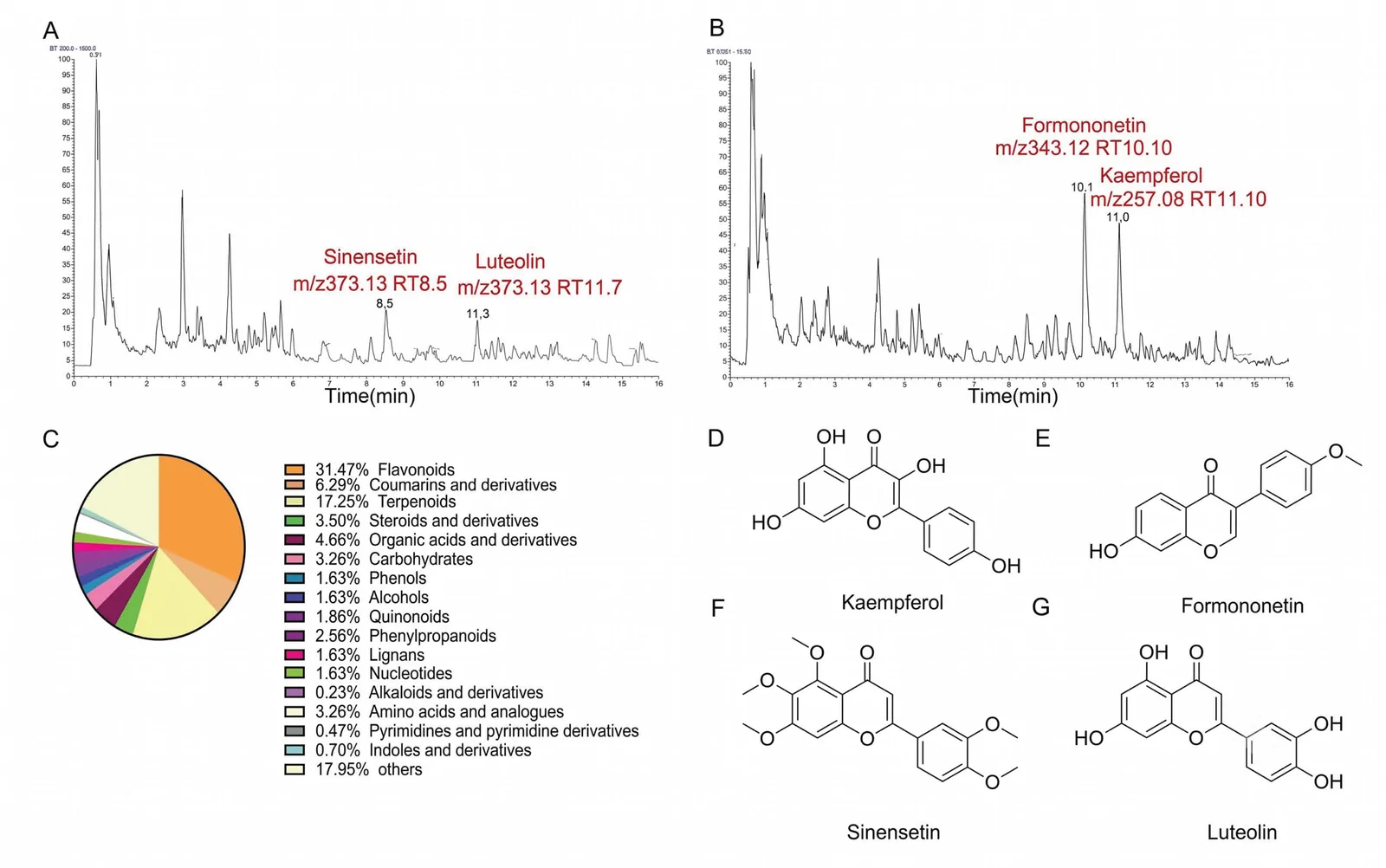
UPLC/MS-MS analysis of SLBZS. (**A**) Total ion chromatogram in negative-ion mode of SLBZS. (**B**) Total ion chromatogram in positive-ion mode of SLBZS. (**C**) Proportion diagram of the component types of SLBZS. (**D-G**) Main Chemical structure of Kaempferol, Formononetin, Sinensetin, and Luteolin.

**Table 1:** The composition of SLBZS.

**Taxonomy Name**	**Chinese Name**	**Used Part**	**Weight (g)**
*Codonopsis pilosula (Franch.) Nannf*	Dang Shen	Root	20g
*Poria cocos(Schw.)Wolf*	Fu Ling	Sclerotium	15g
*Atractylodes macrocephala Koidz*	Bai Zhu	Rhizome	20g
*Dioscorea opposite Thunb.*	Shan Yao	Rhizome	20g
*Dolichos lablab L.*	Bai Bian Dou	Seed	15g
*Semen Nelumbinis.*	Lian Zi	Seed	10g
*Glycyrrhiza uralensis Fisch.*	Gan Cao	Root and rhizome	10g
*Coix lacryma-jobi L.*	Yi Yi Ren	Seed	10g
*Amomum villosum Lour.*	Sha Ren	Fruit	10g
*Platycodon grandifloras.*	Jie Geng	Rhizome	10g

**Note:** The plant name has been checked with http://www.theplantlist.org.

## Data Availability

The data underlying this study's findings can be obtained from the corresponding author upon reasonable request.

## LIST OF ABBREVIATIONS

AhR: Aryl hydrocarbon Receptor
ARA: Arachidonic Acid
DAI: Disease Activity Index
DSS: Dextran Sulfate Sodium
EETs: Epoxyeicosatrienoic acids
EpFAs: Epoxy Fatty Acids
HETEs: Hydroxyeicosatetraenoic acids
IBD: Inflammatory Bowel Disease
PPI: Protein-Protein Interaction
SIN: Sinensetin
SLBZS: Shen-Ling-Bai-Zhu-San
TCM: Traditional Chinese Medicine
TJs: Tight Junctions
UC: Ulcerative Colitis
UPLC-MS/MS: Liquid Chromatography in conjunction with tandem Mass Spectrometry
