# Supplementary material for: Shen-Ling-Bai-Zhu-San Mitigates Ulcerative Colitis by Enhancing Intestinal Barrier Integrity via the AhR-CYP1A1-NF-κB Signal Pathway
Source: Comb Chem High Throughput Screen. 2025 Sep 30;29(7):1220–34. doi: 10.2174/0113862073425771250828112001 (PMC13539592; doi:10.2174/0113862073425771250828112001)
Supplement: Supplementary file 1 [file CCHTS-29-7-1220_SD1.pdf]

Supplementary Material

Shen-Ling-Bai-Zhu-San Mitigates Ulcerative Colitis by Enhancing Intestinal Barrier Integrity *via* the AhR-CYP1A1-NF-κB Signal Pathway

Lan Ming[1,2], Jie Chen[1,], Jing Ma[1], ShiQi Guo[1], Ke Xu[1], JiaMin Ji[3], ZhiRong Zhao[4], ShuGuang Xu[1,*] and Qian Huang[2,*]

[1]YanchengTCM Hospital Affiliated to Nanjing University of Chinese Medicine, Yancheng 224000, China; [2]Jinling Clinical Medical College, Nanjing University of Chinese Medicine, Nanjing 210000, China; [3]Research Institute of General Surgery, Jinling Hospital, Southeast University, Nanjing 210000, China; [4]Research Institute of General Surgery, Jinling Hospital, Nanjing University, Nanjing 210000, China

SUPPLEMENTARY METHOD S1.

Detailed Experimental Procedures for SLBZS Preparation and UPLC–MS/MS Analysis

Preparation of SLBZS Decoction

SLBZS was prepared following the classical prescription proportions: Codonopsis pilosula(Franch.)Nannf(20g), Poria cocos(Schw.)Wolf(15g), Atractylodes macrocephala Koidz (20g), Dioscorea opposite Thunb. (20g), Dolichos Lablab L.(15g), Semen Nelumbinis.(10g), Glycyrrhiza uralensis Fisch.(10g), Amomum villosum Lour. (10g), Coix lacryma-jobi L.(10g), and Platycodon grandifloras(10g). The herbal mixture was soaked in distilled water (1:10, w/v) for 30 min at room temperature, followed by two successive 30 min reflux extractions with distilled water. The combined extracts were filtered, and the filtrate was concentrated under reduced pressure using a rotary evaporator (RE-52A, Yarong, China) to a final volume of 62 mL. The concentrate was then lyophilized and stored at -20° C until further use.

Sample Preparation for UPLC–MS/MS

An accurately weighed 0.2 g portion of lyophilized SLBZS extract was dissolved in 10 mL methanol and subjected to ultrasonic extraction (40 kHz; KQ-500DE, Kunshan Ultrasonic Instruments, China) for 30 min. The extract was centrifuged at 12,000 rpm for 10 min (H1650-W, Xiangyi, China), and the resulting supernatant was filtered through a 0.22 μm membrane (Millipore, USA). Samples were diluted appropriately to fall within the calibration curve's linear range prior to injection.

UPLC–MS/MS Conditions

Analyses were conducted on a Thermo Scientific™ Q Exactive™ Hybrid Quadrupole–Orbitrap Mass Spectrometer (Thermo Fisher Scientific, USA) equipped with a Waters ACQUITY UPLC BEH C18 column (2.1 × 100 mm, 1.7 μm) maintained at 40 °C. The mobile phases consisted of 0.1% formic acid in water (A) and acetonitrile (B). The flow rate was set at 0.5 mL/min with a 3 μL injection volume. The gradient elution program was as follows: 0–11 min, 85% A to 25% A; 11–12 min, 25% A to 2% A; 12–14 min, hold at 2% A; 14–14.1 min, 2% A to 85% A; and 14.1–16 min, hold at 85% A.

Mass Spectrometry Parameters

The sheath and auxiliary gas flow rates were 50 and 13 Arb, respectively. The ion transfer tube and vaporizer temperatures were maintained at 320 °C and 350 °C, respectively. Spray voltages were set to 3.5 kV in positive ion mode and 3.0 kV in negative ion mode. Full MS resolution was 70,000, and MS/MS resolution was 17,500, with normalized collision energies of 20/40/60. MS and MS/MS spectra were matched against the proprietary TCM metabolite database (MJBIOTCM, Shanghai Majorbio Bio-Pharm Technology Co., Ltd.).

Quantification of Major Constituents

Reference standards of sinensetin, kaempferol, formononetin, and luteolin (purity ≥ 98%, Shanghai Yuanye Bio-Technology Co., Ltd.) were prepared in methanol at concentrations ranging from 1–500 ng/mL to construct calibration curves ($R^2 > 0.996$). The concentrations of these compounds in SLBZS were determined using the external standard method and expressed as μg/g of dry extract. All measurements were conducted in triplicate to ensure reproducibility.

SUPPLEMENTARY METHOD S2.

Network Pharmacology Analysis

SLBZS components were identified via UPLC–MS/MS and matched to protein targets in the TCMSP database (https://old.tcmsp-e.com/tcmsp.php) using oral bioavailability (OB) ≥ 30% and drug-likeness (DL) ≥ 0.18 as screening thresholds. Target names were standardized to official gene symbols through the UniProt database (https://www.uniprot.org/). UC-associated targets were retrieved from the GeneCards database (https://www.genecards.org/), and only those with relevance scores above the median quartile were retained. Overlapping targets between SLBZS and UC were identified using the Venny 2.1 tool (https://bioinfogp.cnb.csic.es/tools/venny/).

A protein–protein interaction (PPI) network of the shared targets was constructed using the STRING database (https://string-db.org/) with a minimum interaction score of 0.4 (medium confidence) and visualized in Cytoscape 3.9.1. Functional enrichment analyses, including Gene Ontology (GO) and Kyoto Encyclopedia of Genes and Genomes (KEGG) pathway analysis, were performed using DAVID (https://david.ncifcrf.gov/) and Metascape (https://metascape.org/), with statistical thresholds set at $P < 0.05$ and enrichment factor > 1.5. Visualization of enrichment results was carried out via the MicroBiotech platform (https://www.microbiotech.com/).

SUPPLEMENTARY METHOD S3.

Molecular docking

Four representative bioactive compounds—sinensetin (CID: 5320940), kaempferol (CID: 5280863), formononetin (CID: 5280378), and luteolin (CID: 5280445)-were selected as ligands, with their 3D structures downloaded from the PubChem database (https://pubchem.ncbi.nlm.nih.gov/) and energy-minimized using AutoDockTools. Protein structures of CYP1A1 (PDB ID: 4I8V), AHR (PDB ID: 5NJ8), and NF-κB1(PDB ID:1NFK) were obtained from the Protein Data Bank (https://www.rcsb.org/). Prior to docking, all water molecules were removed, polar hydrogens were added, and Gasteiger charges were assigned using AutoDockTools.

Docking simulations were performed with AutoDock Vina, setting grid box dimensions to fully encompass the active site of each target protein and a grid spacing of 1.0 Å. Binding affinities equal to or less than −5.0 kcal/mol were considered indicative of stable ligand–receptor interactions. The docking results were visualized and analyzed using PyMOL to evaluate binding poses and interaction patterns.

Table 1. Criteria for disease activity index.

Weight loss (%)	Stool consistency	Blood loss	Score
None	Normal droppings	None	0
1-5			1
6-10	Loose droppings	Mild bleeding	2
11–15			3
> 15	Diarrhea	Gross bleeding	4

Table 2. Active components of SLBZS.

Number	m/z	Molecule Name	Classification	Row Mean
1	373.1281872	Luteolin	Flavonoids	125260502.7
2	257.0807507	Kaempferide	Flavonoids	35713140.54
3	373.1281707	Sinensetin	Flavonoids	33576264.53
4	343.1175776	Formononetin	Flavonoids	33024223.5
5	453.3363511	18 beta-Glycyrrhetintic Acid	Terpenoids	31570786.78
6	341.1087627	Sucrose	Carbohydrates	30648027.25
7	389.1231062	5-O-Demethylnobiletin	Flavonoids	24639061.26
8	373.1280659	Isosinensetin	Flavonoids	24573729.15
9	431.1335999	Formononetin glucoside	Flavonoids	21448055.57
10	549.161521	Liquiritin apioside	Flavonoids	19008607.8

11	343.1176129	6-Demethoxytangeretin	Flavonoids	14573595.19
12	285.0757596	Acacetin	Flavonoids	14364196.2
13	269.0807658	Tectochrysin	Flavonoids	12125431.01
14	257.0807711	Liquiritigenin	Flavonoids	11518125.36
15	165.054587	P-hydroxylphenylpropanol	Organic acids and derivatives	11378219.08
16	419.1335729	Isoliquiritin	Flavonoids	11264270.7
17	511.3420051	Alisol C 23-acetate	Terpenoids	11210639.96
18	455.3520484	Beta-Elemonic acid	Terpenoids	10925383.37
19	609.182645	Neohesperidin	Flavonoids	10608556.4
20	433.149444	3',4',3,5,6,7,8-Heptamethoxyflavone	Flavonoids	9954050.973
21	369.1334747	Glycycoumarin	Flavonoids	8941398.37
22	469.3313906	Quillaic acid	Terpenoids	8039970.397
23	144.1020865	Stachydrine	Amino acids and analogues	7610402.299
24	116.0709327	2-Pyrrolidinecarboxylic acid	Amino acids and analogues	7050582.289
25	357.0980303	Ophiopogonanone H	Others	6438873.006
26	611.1970835	Hesperidin	Flavonoids	6263010.336
27	283.0611951	Calycosin	Flavonoids	5896015.637
28	268.1042166	Adenosine	Nucleotides	5828325.465
29	255.0660805	Dihydrodaidzein	Flavonoids	5762177.678
30	455.3521163	Dehydrotrametenolic acid	Terpenoids	5740160.831
31	389.1230625	Artemetin	Flavonoids	5723511.516
32	369.1333218	Anhydroicaritin	Flavonoids	5604218.823
33	549.161635	Isoliquiritin apioside	Flavonoids	5540134.121
34	661.3949537	27-Deoxyactein	Steroids and derivatives	5298931.29
35	187.0391467	Psoralen	Coumarins and derivatives	5156200.335
36	269.0806479	5,7,8,4'-Tetramethoxyflavone	Flavonoids	4974131.022
37	303.0862638	Apigenin	Flavonoids	4819999.195
38	825.4649759	Saikosaponin A	Terpenoids	4713357.626
39	177.0547247	Isoferulic acid	Organic acids and derivatives	4490497.859
40	177.0547675	Dimethyl phthalate	Others	4280831.283
41	373.1281872	Tangeretin	Flavonoids	125260502.7
42	495.3468632	Dehydroalisol B 23-acetate	Others	4257152.822
43	195.0501039	D-Gluconic acid	-	3987068.946
44	375.1074231	Casticin	Flavonoids	3755549.189
45	271.0964458	Retrochalcone	Flavonoids	3668230.612
46	423.1803756	Mulberrin	Flavonoids	3484185.167
47	871.4700859	Cyclocephaloside II	Steroids and derivatives	3216723.611
48	463.1598586	(glucopyanoside	-	3216540.371
49	595.1661735	Vicenin II	Flavonoids	3137782.089

50	207.1016359	Senkyunolide H	Others	3000390.219
51	301.1069811	Methylnissolin	Flavonoids	2748188.776
52	763.4625976	Saikosaponin B1	Terpenoids	2703490.739
53	437.3413743	Alisol B	Terpenoids	2683019.274
54	829.4597225	Astragaloside III	Steroids and derivatives	2607978.219
55	187.0390942	Isopsoralen	Coumarins and derivatives	2533072.807
56	177.0549338	Ferulic acid	Organic acids and derivatives	2462383.569
57	437.3412498	Corosolic acid	Terpenoids	2305138.321
58	471.3472326	Glycyrrhetic acid	Terpenoids	2286188.735
59	439.3571214	Ursolic acid	Terpenoids	2047069.843
60	645.3648323	Glycyrrhetic acid 3-O-mono-b-D-	-	1949397.153
61	127.0392912	5-Hydroxymethyl-2-Furaldehyde	Others	1945222.952
62	453.3362799	11-Keto-beta-boswellic acid	Terpenoids	1929297.591
63	177.0547578	Caffeic Acid Methyl Ester	Organic acids and derivatives	1921413.095
64	339.1236422	Dehydrodiconiferylalcohol	Flavonoids	1860235.227
65	323.0982788	3-Hydroxy-3-methylglutarate	-	1854059.677
66	130.0865029	Pipecolinic acid	Amino acids and analogues	1843699.171
67	285.0755307	Glycitein	Flavonoids	1641248.862
68	909.5201478	Saikosaponin bk1	Terpenoids	1603702.62
69	871.4697163	Astragaloside II	Steroids and derivatives	1595119.018
70	127.0393181	5-Hydroxymethylfurfural	Others	1548391.923
71	425.1966508	Beta-Mangostin	Others	1518069.479
72	437.341488	Hederagenin	Terpenoids	1510061.2
73	130.0501475	L-Glutamic Acid	Amino acids and analogues	1500374.693
74	339.1591075	Bavachinin	Flavonoids	1500123.956
75	179.0339876	Caffeic acid	Organic acids and derivatives	1479536.065
76	623.1619644	2''-O-Rhamnosylvitexin	Flavonoids	1447875.148
77	207.10171	Senkyunolide I	Others	1409749.78
78	345.0969436	Lysionotin	Flavonoids	1404937.763
79	455.3520304	Alisol G	Terpenoids	1370392.216
80	455.352024	Echinocystic acid	Terpenoids	1352070.134
81	359.1126498	Gardenin B	Flavonoids	1323818.674
82	136.0620189	Adenine	Others	1318109.82
83	437.3413941	3-Epicorosolic acid	Terpenoids	1292607.619
84	489.139001	6''-O-Acetylglycitin	Flavonoids	1269910.432
85	167.0339173	Homogentisic acid	-	1245327.385
86	515.1194364	Isochlorogenic acid B	Alcohols	1236821.376
87	913.4804048	Dioscin	Steroids and derivatives	1227566.965
88	175.0602195	2-Isopropylmalic acid	-	1179573.278

89	563.1407747	Vicenin-1	Flavonoids	1152392.892
90	577.1564948	Violanthin	Flavonoids	1151084.283
91	463.1613601	Isomucronulatol 7-O-glucoside	Flavonoids	1133490.079
92	301.0705989	Tectorigenin	Flavonoids	1089323.584
93	373.0927359	Chrysosplenetin B	Flavonoids	1077124.698
94	337.1082101	Licoflavone C	Flavonoids	1061942.291
95	491.1196084	Calycosin-7-O-beta-D-glucoside	Flavonoids	1049935.316
96	353.0879186	1-O-Caffeoylquinic acid	Alcohols	1043344.539
97	124.0396291	Nicotinic acid	Others	1026819.848
98	355.1538995	Licochalcone D	Flavonoids	1022129.546
99	487.3420857	Alisol C	Terpenoids	968729.4254
100	287.0913973	Sappanchalcone	Phenylpropanoids	960183.76
101	515.2275357	Nomilin	Terpenoids	955932.478
102	182.0814579	L-Tyrosine	Amino acids and analogues	955032.4916
103	482.2021771	Isomucronulatol-7-O-beta-glucoside	Flavonoids	936281.8624
104	233.1537369	Atractylenolide II	Terpenoids	927122.6738
105	823.410682	Glycyrrhizic acid	Terpenoids	920364.7648
106	284.0992162	Crotonoside	Nucleotides	913632.0228
107	118.0865081	Betaine	Amino acids and analogues	900791.1948
108	271.060133	4',6,7-Trihydroxyisoflavone	Flavonoids	895889.6533
109	355.1541228	Xanthohumol	Flavonoids	881833.999
110	463.1233933	Isoscoparin	Flavonoids	881673.9954
111	447.0920679	Baicalin	Flavonoids	866864.8635
112	131.0449272	L-Asparagine	Amino acids and analogues	854032.9712
113	299.0913512	Moslosooflavone	Flavonoids	847694.4272
114	469.3314167	Glabrolide	Terpenoids	839638.6187
115	287.0913015	Licochalcone B	Flavonoids	772642.505
116	137.0231461	4-Hydroxybenzoic acid	Others	747830.4976
117	215.0320463	D-Mannose	Carbohydrates	739399.3602
118	423.3623133	Roburic acid	Terpenoids	701020.5117
119	353.1030112	Sanggenon H	Flavonoids	672409.1763
120	343.0821731	Usnic acid	Others	660259.8272
121	419.1338212	Liquiritin	Flavonoids	659593.9459
122	273.0757181	Naringenin chalcone	Flavonoids	641281.4746
123	946.5729216	Saikosaponin F	Terpenoids	627444.0235
124	299.0560268	3'-Methoxyapigenin	Flavonoids	625654.9029
125	369.1334991	Icaritin	Flavonoids	611704.7989
126	595.2021763	Poncirin	Flavonoids	603182.8092
127	301.0717057	Hesperetin	Flavonoids	598913.0881

128	503.1618645	1-Kestose	Carbohydrates	596695.0097
129	181.0707621	Dulcitol	Carbohydrates	584046.7878
130	355.1178402	Licoflavonol	Flavonoids	582735.5934
131	453.3365378	Asiatic acid	Terpenoids	552309.937
132	269.0455526	Emodin	Quinonoids	542950.1292
133	243.0618268	Uridine	Nucleotides	536546.0017
134	439.3570512	(+)-Ganodermanondiol	Terpenoids	531751.075
135	339.1228577	Wighteone	Flavonoids	525430.0781
136	419.1351516	4-deoxyphloridzin	-	519769.187
137	147.0286863	(-)-Citramalic acid	-	515957.0603
138	191.055204	D-(-)-Quinic acid	Alcohols	504254.8434
139	144.0658137	Maltol	Others	501535.3967
140	223.0602253	Isofraxidin	Coumarins and derivatives	501114.1173
141	891.4722915	Isoastragaloside I	Steroids and derivatives	500706.8492
142	301.070605	Hispidulin	Flavonoids	499994.0919
143	941.5114469	Oplopanaxoside D	Terpenoids	494577.1508
144	135.1169648	(-)-Fenchone	Terpenoids	494001.4468
145	253.0503889	Daidzein	Flavonoids	486054.0634
146	397.2018975	Arenobufagin	Steroids and derivatives	481266.5076
147	943.5255325	Soyasaponin Bb	Terpenoids	478558.3106
148	191.1068503	Alpha-Asarone	Others	477634.8511
149	275.200613	Nandrolone	Steroids and derivatives	470078.6635
150	445.0777365	Glychionide	-	470072.6679
151	137.0231641	Salicylic Acid	Others	465794.2722
152	173.0081134	Citric acid	Organic acids and derivatives	463666.308
153	603.3893418	Cimigenol-3-O-beta-D-xylpyranoside	Steroids and derivatives	456665.4136
154	285.0757887	Biochanin A	Flavonoids	454855.2635
155	253.0503738	Chrysin	Flavonoids	447898.7735
156	609.1462808	Rutin	Flavonoids	444356.5337
157	609.1810973	Diosmin	Flavonoids	439997.272
158	207.0652291	Citropten	Coumarins and derivatives	436045.3852
159	167.0704175	O-Veratraldehyde	Others	435684.0541
160	299.0915093	Dihydromethysticin	Phenylpropanoids	433521.5913
161	461.1440283	glucopyranoside	Flavonoids	424641.5809
162	152.0567803	Guanine	Others	422223.5214
163	254.2479023	Ambroxane	Others	421314.0718
164	162.0762123	D-Glucosamine	Carbohydrates	417655.1448
165	469.3321908	16alpha-	Terpenoids	415900.6959
166	112.0509235	Cytosine	Pyrimidines and pyrimidine	412694.9471

167	207.0653039	Scoparone	Coumarins and derivatives	409034.4965
168	163.0390135	4-Coumaric acid	-	408490.8605
169	487.3420208	Madecassic acid	Terpenoids	400134.9716
170	123.0556091	Nicotinamide	Others	395062.0832
171	414.2121748	Lobetyolin	Others	391257.4139
172	148.1122577	Perillartine	Terpenoids	377355.2269
173	581.1862291	Narirutin	Flavonoids	375790.4129
174	163.1231894	L-Nicotine	Others	367385.8773
175	461.1079044	Oroxylin A-7-O-beta-D-glucuronide	Flavonoids	361700.6543
176	282.084342	Guanosine	Nucleotides	360617.2037
177	287.0913446	Protosappanin B	Others	350589.7094
178	151.0392014	Griffonilide	Others	348853.7859
179	355.2230219	Periplogenin	Steroids and derivatives	336594.65
180	497.3623925	Bryodulcosigenin	Steroids and derivatives	329379.2994
181	133.1013661	Perillene	Others	327216.1546
182	231.137971	Atractylenolide III	Terpenoids	326468.7985
183	171.1016062	Azelaic acid	Others	323674.389
184	107.0496328	P-hydroxylbenzyl alcohol	Others	320996.1934
185	439.3571258	Oleanolic acid	Terpenoids	320004.3241
186	164.0706684	L-(-)-Phenylalanine	-	319026.3835
187	335.092503	Psoralidin	Flavonoids	310493.1171
188	151.0390661	4-Methoxysalicylic acid	Others	310455.9279
189	623.161807	Narcissoside	Flavonoids	308160.9489
190	341.1386283	8-Prenylnaringenin	Flavonoids	305021.072
191	491.1197774	IristectorinA	Flavonoids	304054.2282
192	989.3199343	1,1,1,1-Kestohexaose	Carbohydrates	303769.1494
193	825.4642086	Saikosaponin B2	Terpenoids	301993.3299
194	163.0754421	5-Hydroxy-1-tetralone	Others	301380.0511
195	665.2149004	Stachyose	Carbohydrates	299351.7559
196	273.0757641	Naringenin	Flavonoids	299310.5477
197	135.0439056	4'-Hydroxyacetophenone	Others	299139.9583
198	203.0818969	L-Tryptophan	Indoles and derivatives	292900.087
199	403.1387177	Nobiletin	Flavonoids	292374.3381
200	463.1234368	Tectoridin	Flavonoids	291167.42
201	113.0349343	Uracil	Pyrimidines and pyrimidine	286246.1169
202	330.0597725	Adenosine Cyclophosphate	Nucleotides	281828.6682
203	415.1036226	Daidzin	Flavonoids	280903.3258
204	170.0813431	Vitamin B6	Others	277083.3571
205	161.0600016	Cantharidin	Others	274697.6107

206	189.1122177	Eucommiol	Terpenoids	273088.3152
207	398.232682	Levistilide A	Others	272506.7481
208	433.112891	Vitexin	Flavonoids	266742.9792
209	355.1186257	8-Formylmethylophiopogonanone B	Flavonoids	265554.2117
210	361.0917154	Irigenin	Flavonoids	264966.2779
211	147.0442904	Coumarin	Coumarins and derivatives	261100.8288
212	133.1013346	Thymol	Terpenoids	260126.5392
213	447.0934818	Homoorientin	Flavonoids	259838.5686
214	595.1656024	Lonicerin	Flavonoids	254649.947
215	331.0811878	Aurantio-obtusin	Quinonoids	248439.2416
216	161.05993	6-Methylcoumarin	Coumarins and derivatives	241497.8536
217	805.4731355	6''-O-Acetylsaikosaponin D	Terpenoids	240172.509
218	147.0441308	P-Coumaric acid	Organic acids and derivatives	239863.4063
219	299.0559602	Isoliquiritigenin	Flavonoids	239645.9332
220	513.3579248	Alisol A	Terpenoids	236223.9248
221	503.1909154	Pinoresinol 4-O-glucoside	Lignans	230247.3305
222	299.0560451	Diosmetin	Flavonoids	226160.9279
223	343.0821396	Eupatilin	Flavonoids	224258.1933
224	445.1139136	Physcion 8-O-beta-D-monoglucoside	Quinonoids	213969.1391
225	163.0755811	Methyl cinnamate	Organic acids and derivatives	212985.4386
226	435.129746	Phloridzin	Flavonoids	212919.0612
227	809.4692571	Saikosaponin E	Terpenoids	212002.7328
228	269.0454518	Galangin	Flavonoids	211062.2558
229	153.0181708	Gentisic acid	Others	210135.9045
230	499.1234052	1,3-Dicaffeoylquinic acid	Alcohols	209038.0308
231	283.061117	Prunetin	Flavonoids	207830.6877
232	355.0824241	Methyl rosmarinate	Organic acids and derivatives	207498.7343
233	819.4304724	Astragaloside IV	Steroids and derivatives	207263.5195
234	151.059946	Xylitol	Carbohydrates	207050.7745
235	385.1130258	Sesamoside	Carbohydrates	206257.9603
236	485.3259853	Poricoic acid B	Terpenoids	205991.7703
237	615.1491277	Naringin	Flavonoids	204995.2579
238	331.0811753	Jaceosidin	Flavonoids	203511.9339
239	271.0600137	Aloeemodin	Quinonoids	196750.0437
240	285.0758311	Galangin 3-methyl ether	Flavonoids	196173.6914
241	131.0493586	Cinnamic acid	Organic acids and derivatives	193055.4091
242	251.1645129	Nardosinone	Terpenoids	192511.5307
243	433.1130851	Apigenin-7-glucoside	Flavonoids	191044.968
244	121.0651396	Acetophenone	Others	190936.7713

245	344.0399318	Guanosine-5'-monophosphate	Nucleotides	190606.4131
246	191.1067888	Ligustilide	Others	188075.6501
247	259.0964418	Lunularic acid	Phenylpropanoids	187378.7739
248	287.0560792	(+)-Gallocatechin	Flavonoids	187327.8457
249	137.0598448	Anisaldehyde	Others	185453.385
250	421.1649209	Kuwanon A	Flavonoids	184364.2456
251	329.101954	Salvigenin	Flavonoids	181860.7028
252	137.0598306	Anisic aldehyde	Others	180624.1461
253	269.0455523	Baicalein	Flavonoids	180160.2649
254	299.0561005	Hydroxygenkwanin	Flavonoids	178878.8716
255	633.4002902	Kajiichigoside F1	Terpenoids	178484.2205
256	252.109312	Cordycepin	Nucleotides	177175.2124
257	629.1647293	2',6'-Dihydroxy-4-Methoxychalcone-4'-O-Neohesperidoside	-	176202.7376
258	387.1074715	Moluccanin	Lignans	174627.9032
259	191.1068414	3-n-Butylphathlide	Others	174213.3543
260	149.044307	D-Ribose	Carbohydrates	172683.456
261	387.1090107	Methylophiopogonanone A	Flavonoids	171678.9138
262	473.3628365	Maslinic acid	Terpenoids	168548.501
263	513.358264	Acetyl-11-keto-beta-boswellic acid	Terpenoids	167120.474
264	191.1067477	Cis-beta-asarone	Others	166635.8567
265	421.1648336	Morusin	Flavonoids	163142.6527
266	201.1124456	Sebacic acid	-	162892.3314
267	449.1080011	Orientin	Flavonoids	162693.1897
268	471.2012929	Limonin	Terpenoids	161673.8825
269	313.0706999	Aurantio-obtusifolin	-	160493.5878
270	207.0655328	Sinapic acid	Organic acids and derivatives	156919.7494
271	449.1442545	Agnuside	Terpenoids	156434.9886
272	455.3522424	Ganodermanontriol	Terpenoids	154761.6453
273	313.071863	Pectolinarigenin	Flavonoids	154644.8373
274	193.0862541	Myristicin	Others	152938.1791
275	151.038904	Methyl 4-hydroxybenzoate	Others	147538.4424
276	247.0601033	Pimpinellin	Coumarins and derivatives	146294.6993
277	207.0653274	Lawsone	Quinonoids	144409.5119
278	324.2897531	Linolenic acid ethyl ester	Others	143915.7679
279	161.059838	Ferulaldehyde	Phenols	142556.6224
280	131.0494631	2-Hydroxycinnamaldehyde	Phenylpropanoids	141828.6941
281	329.066712	Tricin	Flavonoids	141432.8729
282	321.113089	Glabrene	Flavonoids	141130.6321

283	317.0655342	Isorhamnetin	Flavonoids	140542.1682
284	193.1225091	Senkyunolide	Others	139886.2793
285	193.0497304	Noreugenin	Others	139317.0078
286	192.0658047	3-Furfuryl 2-pyrrolecarboxylate	Others	138293.095
287	525.1615269	Albiflorin	Terpenoids	138117.3217
288	153.0182115	Protocatechuic acid	Others	136379.3404
289	177.0183862	Aesculetin	Coumarins and derivatives	134693.6397
290	377.1456486	Vitamin B2	Others	133713.2068
291	273.0756874	Pinobanksin	Flavonoids	132900.4747
292	299.091325	5-Hydroxy-6,7-dimethoxylflavone	Flavonoids	132764.9348
293	153.054805	Vanillin	Phenols	132007.6596
294	499.1233136	3,5-Di-O-caffeoylquinicacid	-	128289.0538
295	265.1436364	Abscisic acid	Terpenoids	125761.1975
296	273.1848647	Estradiol	Steroids and derivatives	123166.8759
297	177.0394419	D(-)-Gulono-gamma-lactone	-	121607.3966
298	156.0769533	L-Histidine	Amino acids and analogues	121067.4409
299	112.0759788	4-Hydroxyisoleucine	Amino acids and analogues	119463.9756
300	339.1591205	Licochalcone A	Flavonoids	118848.3244
301	439.3571208	Alpha-Boswellic acid	Terpenoids	117475.0265
302	461.1078627	Wogonin 7-O-glucuronide	Flavonoids	117074.5753
303	291.0863245	Bilobalide	Terpenoids	115214.1152
304	171.0654637	Protocatechuic aldehyde	Others	113369.9253
305	312.124164	N-trans-Feruloyltyramine	Phenols	113028.95
306	385.1136464	1-O-b-D-Glucopyranosyl sinapate	-	112900.9547
307	123.0444008	P-Hydroxybenzaldehyde	Others	111890.485
308	357.1343309	Matairesinol	Lignans	111651.2123
309	303.1227985	Isomucronulatol	Flavonoids	110694.8616
310	255.0653598	Chrysophanol	Quinonoids	110662.0252
311	273.0768707	Phloretin	Flavonoids	109253.1047
312	159.0918753	5-Hydroxytryptamine	Indoles and derivatives	108832.0646
313	169.0498294	Vanillic acid	Others	105913.7872
314	487.3421384	16-Oxoalisol A	Terpenoids	105489.383
315	193.0499028	4-Methyl-6,7-dihydroxycoumarin	Coumarins and derivatives	105099.9202
316	359.0770633	5,7,3'-Trihydroxy-6,4',5'-trimethoxyflavone	Flavonoids	104060.543
317	218.1027547	Pantothenate	-	102713.8937
318	315.0511777	Rhamnetin	-	102404.3172
319	375.1074722	Vitexicarpin	Flavonoids	101994.1579
320	327.1085649	Aucubin	Terpenoids	101987.6302
321	123.0443119	Benzoic acid	Others	101159.0594

322	193.0497425	5,7-Dihydroxy-4-methylcoumarin	Coumarins and derivatives	100947.0316
323	149.0598343	Ethylparaben	Others	99582.61092
324	191.070366	7-Methoxy-4-methylcoumarin	Coumarins and derivatives	99189.75985
325	257.0816267	Rhapontigenin	Phenylpropanoids	98834.73943
326	227.1030014	S-(-)-Carbidopa	Organic acids and derivatives	98408.36763
327	201.1275998	Pterosin B	Others	96985.60756
328	137.132619	Linalool	Terpenoids	95633.96553
329	225.0609716	Inositol	Alcohols	93081.55184
330	215.1067538	Fraxinellone	Others	93034.96107
331	421.3468912	11-Deoxyalisol B	Terpenoids	91298.34292
332	287.055018	Tangeretin	Flavonoids	91218.1591
333	401.1595044	(+)-Syringaresinol	Lignans	90298.29218
334	497.3625058	3-O-Acetyl-1alpha-hydroxytrametenolic acid	Terpenoids	90259.65238
335	209.1172699	Elemicin	Others	89741.89068
336	271.1693474	Estrone	Steroids and derivatives	89454.02116
337	195.1382091	Neocnidilide	Others	87526.4307
338	161.059972	4-Methoxycinnamic acid	Organic acids and derivatives	86375.17234
339	181.0497196	DL-p-Hydroxyphenyllactic acid	-	86330.23129
340	463.1238315	Homoplantaginin	Flavonoids	86192.81893
341	303.0500319	Morin	Flavonoids	85287.14577
342	449.1090903	Flavanomarein	-	85067.5282
343	169.0498096	Orsellinic acid	Others	84489.12904
344	339.1228134	Demethoxycurcumin	Phenylpropanoids	83171.19453
345	389.1595066	Trachelogenin	Lignans	83060.46861
346	483.3108958	Ganoderic acid C2	Terpenoids	83032.40788
347	269.0819246	Medicarpin	Flavonoids	82713.13163
348	466.2652853	Atractyloside A	Terpenoids	82654.23518
349	325.1434825	Isobavachalcone	Flavonoids	82361.53791
350	353.0879214	Chlorogenic Acid	Alcohols	82195.82934
351	374.1450165	Gentiopicrin	Carbohydrates	81867.28842
352	195.1017293	2-Methoxycinnamaldehyde	Phenylpropanoids	81790.31137
353	287.0550421	Fisetin	Flavonoids	80571.3997
354	307.1025685	D-(+)-Lactose	Carbohydrates	79692.80672
355	481.2947251	Ganoderic acid B	Terpenoids	78980.23533
356	193.1225334	Senkyunolide A	Others	78781.07361
357	301.0352763	Quercetin	Flavonoids	77665.02656
358	385.1294815	Wushanicaritin	Flavonoids	77338.58067
359	197.0447598	Danshensu	Organic acids and derivatives	77283.97523

360	387.1043215	Irisflorentin	Flavonoids	77216.36075
361	285.040493	3-Hydroxymorindone	Quinonoids	76775.44062
362	169.0132423	Gallic acid	Others	74677.29332
363	329.138433	Decursin	Coumarins and derivatives	73744.60359
364	137.0460025	Hypoxanthine	Others	73228.96145
365	467.316453	Ganoderic acid DM	Terpenoids	72696.29849
366	283.0611223	Wogonin	Flavonoids	72112.55635
367	217.1224104	Pterosin G	Others	71364.31733
368	223.0599961	Fraxinol	Coumarins and derivatives	71210.64551
369	307.1173951	Cimifugin	Others	70664.95167
370	315.0497248	Wedelolactone	Flavonoids	70308.19157
371	551.33472	23-Acetyl alisol C	Terpenoids	69590.32353
372	209.0809764	4-Methylumbelliferone	Coumarins and derivatives	69121.06538
373	219.1744147	Curcumol	Terpenoids	69057.67615
374	249.1485404	Parthenolide	Terpenoids	67687.57832
375	355.1034765	Geniposidic acid	Terpenoids	66725.57699
376	371.1488219	Eurycomalactone	Steroids and derivatives	66680.38724
377	273.0758603	(+)-Catechin	Flavonoids	65152.55572
378	315.1227207	Flavokawain A	Flavonoids	64168.55271
379	247.1329823	Arglabin	Terpenoids	61313.68432
380	193.0499823	Isoscopoletin	Coumarins and derivatives	61163.94748
381	209.0447555	Fraxetin	Coumarins and derivatives	61016.7595
382	317.1018678	Byakangelicin	Coumarins and derivatives	60696.8917
383	285.0758474	Genkwanin	Flavonoids	60246.05882
384	197.1288165	Cyclo-(Leu-Thr)	-	60200.55266
385	453.1918962	Obacunone	Terpenoids	58055.68925
386	361.1657895	Secoisolariciresinol	Lignans	57646.34891
387	215.0320221	D-(+)-Galactose	Carbohydrates	56883.68198
388	135.0442165	3-Methoxybenzoic acid	Others	56346.2948
389	285.0405136	Kaempferol	Flavonoids	56334.59869
390	133.0651142	Cinnamaldehyde	Phenylpropanoids	55569.42172
391	118.0865778	L-Valine	Amino acids and analogues	55178.7926
392	235.1692402	Artemisinic acid	Terpenoids	54846.40239
393	355.1540458	Isoxanthohumol	Flavonoids	54101.98813
394	153.0546637	2-Hydroxy-4-methoxybenzaldehyde	Phenols	53168.70377
395	284.1280894	Paprazine	Organic acids and derivatives	52351.83589
396	199.0605571	Syringic acid	Others	51917.78592
397	287.0561348	Aromadendrin	Flavonoids	51869.05922
398	285.0404101	Taxifolin	Flavonoids	50683.56629

399	179.1066569	1,2-Dimethoxy-4-(1-propenyi)benzene	-	50335.30225
400	251.200682	Germacrone	Terpenoids	49101.52273
401	279.2327394	Linoleic acid	Others	49064.21098
402	161.0598559	7-Methylcoumarin	Coumarins and derivatives	48858.94302
403	191.034074	Scopoletin	Coumarins and derivatives	48026.69674
404	171.0805296	3-Butylidenephthalide	Coumarins and derivatives	47856.43952
405	305.1758111	Falcarindiol	Others	47831.23785
406	288.1230267	Lycorine	Alkaloids and derivatives	46636.21061
407	177.0184266	Daphnetin	Coumarins and derivatives	46009.68172
408	421.1646957	Auriculasin	Flavonoids	45929.51137
409	335.0924751	Atalantoflavone	Flavonoids	45301.15578
410	153.0548573	2-Methoxybenzoic acid	Others	44697.89454
411	153.127603	Camphor	Terpenoids	44514.26324
412	154.997409	Trans-Aconitic acid	Organic acids and derivatives	44031.36028
413	321.1124388	Corylin	Flavonoids	43882.72315
414	507.1141935	Pratensein 7-O-beta-D-glucopyranoside	-	42821.08476
415	457.3678163	Epibetulinic acid	Terpenoids	42700.64542
416	113.0601208	Sorbic acid	Others	42291.04491
417	261.1119667	Isomeranzin	Coumarins and derivatives	41437.75776
418	371.1500049	Arctigenin	Lignans	40801.99259
419	229.0496408	3,8-dihydroxy-urolithin	Coumarins and derivatives	40470.03855
420	329.1030162	Acetylshikonin	Quinonoids	40128.43173
421	419.1351148	Rhaponticin	Phenylpropanoids	40057.52058
422	323.1286829	Glabridin	Flavonoids	38718.93871
423	209.0812227	Raspberry ketone	Phenols	35573.68329
424	287.0560489	Eriodictyol	Flavonoids	35500.57972
425	197.0812056	Xanthoxylin	Others	34801.55694
426	231.1380352	Atractylenolide I	Others	33394.14694
427	153.0547043	4-Mthoxysalicylaldehyde	-	33327.00889
428	170.154046	(+/-)-Camphor	Terpenoids	32890.15769
429	253.0714945	Arbutin	Carbohydrates	32755.45791
430	161.0232687	Umbelliferone	Coumarins and derivatives	31803.8326
431	197.0962126	Dihydrokavain	Phenylpropanoids	31794.58795
432	330.133608	N-trans-Feruloyloctopamine	Organic acids and derivatives	30664.07076
433	177.0183331	5,7-Dihydroxychromone	Others	30524.26658
434	237.1849727	Curdione	Terpenoids	29451.93117
435	219.1744877	Alpha-Cyperone	Terpenoids	29360.62006
436	181.0860737	Isopropyl 4-Hydroxybenzoate	Others	29132.4598
437	183.0652488	3hydroxybenzaldehyde	Phenols	27275.60212

438	395.2047465	Andrographolide	Others	26755.73234
439	279.1592101	Dibutyl phthalate	Others	26183.71416
440	134.0601506	6-Hydroxyindole	Indoles and derivatives	25321.74823
441	317.1392849	Zearalenone	-	24712.34603
442	189.0912191	Ethyl 4-methoxycinnamate	Organic acids and derivatives	24689.34726
443	259.0221964	Alpha-D-glucose-1-phosphate	-	24399.19169
444	177.054748	Methyl 4-hydroxycinnamate	Organic acids and derivatives	24250.50013
445	243.1492354	Huperzine A	Others	20629.65692
446	219.1744877	Alpha-Cyperone	Terpenoids	29360.62006
447	245.0809445	Piceatannol	Phenylpropanoids	19496.734
448	173.0445401	Shikimic acid	Alcohols	19099.30532
449	229.0864478	7-Demethylsuberosin	Coumarins and derivatives	18584.78152
450	179.0704973	Xanthoxyline	Others	18326.72869
451	132.0290082	L-Aspartic Acid	Amino acids and analogues	15084.83638
452	114.055374	L-Hydroxyproline	Amino acids and analogues	14592.09725
453	155.0704013	2,6-Dimethoxyphenol	Phenols	14246.86968
454	174.0550961	1-Methoxy-3-carbaldehyde	-	13417.19176
455	120.0657035	L-Threonine	Amino acids and analogues	11089.94857
456	175.1191536	L(+)-Arginine	Amino acids and analogues	8016.211034
457	147.043941	2-Chromanone	Coumarins and derivatives	7978.19929
458	399.1803529	Schisandrol B	Organic acids and derivatives	4173.973024

Table 3. 676 differentially expressed genes between control individual and UC patients.

Serial Number	ID	Gene.Symbol	Description
1.	Q9HC29	NOD2	Nucleotide Binding Oligomerization Domain Containing 2
2.	P01375	TNF	Tumor Necrosis Factor
3.	P05231	IL6	Interleukin 6
4.	A8K7I4	H19	H19 Imprinted Maternally Expressed Transcript
5.	P22301	IL10	Interleukin 10
6.	P52907	IFNG-AS1	IFNG Antisense RNA 1
7.	Q5VWK5	IL23R	Interleukin 23 Receptor
8.	P01584	CYP1A1	Cytochrome P450 Family 1 Subfamily A Member 1
9.	Q3SY69	FOXD2-AS1	FOXD2 Adjacent Opposite Strand RNA 1
10.	P01911	HLA-DRB1	Major Histocompatibility Complex, Class II, DR Beta 1
11.	O00206	TLR4	Toll Like Receptor 4
12.	P01579	IFNG	Interferon Gamma
13.	P05164	MPO	Myeloperoxidase
14.	P10145	CXCL8	C-X-C Motif Chemokine Ligand 8
15.	P08183	ABCB1	ATP Binding Cassette Subfamily B Member 1

16.	P01137	TGFB1	Transforming Growth Factor Beta 1
17.	P18510	IL1RN	Interleukin 1 Receptor Antagonist
18.	O60603	TLR2	Toll Like Receptor 2
19.	P60568	IL2	Interleukin 2
20.	P04637	TP53	Tumor Protein P53
21.	Q3KP66	INAVA	Innate Immunity Activator
22.	Q676U5	ATG16L1	Autophagy Related 16 Like 1
23.	P02741	CRP	C-Reactive Protein
24.	P16410	CTLA4	Cytotoxic T-Lymphocyte Associated Protein 4
25.	P02788	LTF	Lactotransferrin
26.	Q13651	IL10RA	Interleukin 10 Receptor Subunit Alpha
27.	P05112	IL4	Interleukin 4
28.	P19838	NFKB1	Nuclear Factor Kappa B Subunit 1
29.	P01889	HLA-B	Major Histocompatibility Complex, Class I, B
30.	Q9BZS1	FOXP3	Forkhead Box P3
31.	Q16552	IL17A	Interleukin 17A
32.	Q13568	IRF5	Interferon Regulatory Factor 5
33.	P43405	SYK	Spleen Associated Tyrosine Kinase
34.	A1A4Y4	IRGM	Immunity Related GTPase M
35.	P35222	CYP2E2	Cytochrome P450 Family 1 Subfamily EMember 2
36.	P40692	MLH1	MutL Homolog 1
37.	O76082	SLC22A5	Solute Carrier Family 22 Member 5
38.	Q9UIR0	BTNL2	Butyrophilin Like 2
39.	P01350	GAST	Gastrin
40.	Q9NZH6	IL37	Interleukin 37
41.	P35354	PTGS2	Prostaglandin-Endoperoxide Synthase 2
42.	P35225	IL13	Interleukin 13
43.	P49279	SLC11A1	Solute Carrier Family 11 Member 1
44.	O95150	TNFSF15	TNF Superfamily Member 15
45.	Q02817	MUC2	Mucin 2, Oligomeric Mucus/Gel-Forming
46.	Q08334	IL10RB	Interleukin 10 Receptor Subunit Beta
47.	Q9GZX6	IL22	Interleukin 22
48.	O15553	MEFV	MEFV Innate Immunity Regulator, Pyrin
49.	P05113	IL5	Interleukin 5
50.	P40763	STAT3	Signal Transducer And Activator Of Transcription 3
51.	P25445	FAS	Fas Cell Surface Death Receptor
52.	P35228	NOS2	Nitric Oxide Synthase 2
53.	P26927	MST1	Macrophage Stimulating 1
54.	P31749	AKT1	AKT Serine/Threonine Kinase 1

55.	P08246	ELANE	Elastase, Neutrophil Expressed
56.	P01730	CD4	CD4 Molecule
57.	P02768	ALB	Albumin
58.	P15056	BRAF	B-Raf Proto-Oncogene, Serine/Threonine Kinase
59.	P47712	PLA2G4A	Phospholipase A2 Group IVA
60.	Q96PY5	FMNL2	Formin Like 2
61.	Q13485	SMAD4	SMAD Family Member 4
62.	P24158	PRTN3	Proteinase 3
63.	P37231	PPARG	Peroxisome Proliferator Activated Receptor Gamma
64.	O15105	SMAD7	SMAD Family Member 7
65.	P25021	HRH2	Histamine Receptor H2
66.	Q9H257	CARD9	Caspase Recruitment Domain Family Member 9
67.	P19438	TNFRSF1A	TNF Receptor Superfamily Member 1A
68.	P03956	MMP1	Matrix Metallopeptidase 1
69.	Q04206	RELA	RELA Proto-Oncogene, NF-KB Subunit
70.	P51580	TPMT	Thiopurine S-Methyltransferase
71.	Q9H015	SLC22A4	Solute Carrier Family 22 Member 4
72.	Q04864	REL	REL Proto-Oncogene, NF-KB Subunit
73.	Q9Y2R2	PTPN22	Protein Tyrosine Phosphatase Non-Receptor Type 22
74.	Q14116	IL18	Interleukin 18
75.	Q13459	MYO9B	Myosin IXB
76.	P05109	S100A8	S100 Calcium Binding Protein A8
77.	Q9BWF2	MIR21	MicroRNA 21
78.	P33261	CYP2C19	Cytochrome P450 Family 2 Subfamily C Member 19
79.	P21580	TNFAIP3	TNF Alpha Induced Protein 3
80.	Q8TF47	ZFP90	ZFP90 Zinc Finger Protein
81.	P42226	STAT6	Signal Transducer And Activator Of Transcription 6
82.	Q14765	STAT4	Signal Transducer And Activator Of Transcription 4
83.	P12830	CDH1	Cadherin 1
84.	Q8N8D9	CARINH	Colitis Associated IRF1 Antisense Regulator Of Intestinal Homeostasis
85.	P43246	MSH2	MutS Homolog 2
86.	P23219	PTGS1	Prostaglandin-Endoperoxide Synthase 1
87.	P61278	SST	Somatostatin
88.	Q96P20	NLRP3	NLR Family Pyrin Domain Containing 3
89.	P01583	IL1A	Interleukin 1 Alpha
90.	P34932	HSPA4	Heat Shock Protein Family A (Hsp70) Member 4
91.	Q8IV20	LACC1	Laccase Domain Containing 1
92.	O96017	CHEK2	Checkpoint Kinase 2
93.	P05362	ICAM1	Intercellular Adhesion Molecule 1

94.	Q9HC97	GPR35	G Protein-Coupled Receptor 35
95.	Q9NSC7	ST6GALNAC1	ST6 N-Acetylgalactosaminide Alpha-2,6-Sialyltransferase 1
96.	P01920	HLA-DQB1	Major Histocompatibility Complex, Class II, DQ Beta 1
97.	P51677	CCR3	C-C Motif Chemokine Receptor 3
98.	P14780	MMP9	Matrix Metallopeptidase 9
99.	P52701	MSH6	MutS Homolog 6
100.	P01589	IL2RA	Interleukin 2 Receptor Subunit Alpha
101.	O60674	JAK2	Janus Kinase 2
102.	P20648	ATP4A	ATPase H+/K+ Transporting Subunit Alpha
103.	P84022	SMAD3	SMAD Family Member 3
104.	Q9BYZ8	REG4	Regenerating Family Member 4
105.	P48023	FASLG	Fas Ligand
106.	Q8N726	CDKN2A	Cyclin Dependent Kinase Inhibitor 2A
107.	P25054	APC	APC Regulator Of WNT Signaling Pathway
108.	P29460	IL12B	Interleukin 12B
109.	Q4KWH8	MIR31	MicroRNA 31
110.	P55089	UCN	Urocortin
111.	P23771	GATA3	GATA Binding Protein 3
112.	P14778	IL1R1	Interleukin 1 Receptor Type 1
113.	P57739	CLDN2	Claudin 2
114.	O43586	PSTPIP1	Proline-Serine-Threonine Phosphatase Interacting Protein 1
115.	P09038	FGF2	Fibroblast Growth Factor 2
116.	P01133	EGF	Epidermal Growth Factor
117.	P54821	CERNA3	Competing Endogenous LncRNA 3 For MiR-645
118.	Q9Y239	NOD1	Nucleotide Binding Oligomerization Domain Containing 1
119.	P04040	CAT	Catalase
120.	P01909	HLA-DQA1	Major Histocompatibility Complex, Class II, DQ Alpha 1
121.	P11473	VDR	Vitamin D Receptor
122.	P80511	S100A12	S100 Calcium Binding Protein A12
123.	P00734	F2	Coagulation Factor II, Thrombin
124.	Q9UKX5	LINC02605	Long Intergenic Non-Protein Coding RNA 2605
125.	Q9BR39	CDKN2B-AS1	CDKN2B Antisense RNA 1
126.	P12318	FCGR2A	Fc Gamma Receptor IIa
127.	P43897	MIR155	MicroRNA 155
128.	Q01469	LINC01672	Long Intergenic Non-Protein Coding RNA 1672
129.	P43897	NFE2L2	NFE2 Like BZIP Transcription Factor 2
130.	Q04759	PRKCQ	Protein Kinase C Theta
131.	Q9HBE4	IL21	Interleukin 21
132.	P01732	CD8A	CD8 Subunit Alpha

133.	P29965	CD40LG	CD40 Ligand
134.	P13500	CCL2	C-C Motif Chemokine Ligand 2
135.	P00533	EGFR	Epidermal Growth Factor Receptor
136.	P08571	CD14	CD14 Molecule
137.	P10645	CHGA	Chromogranin A
138.	P42898	MTHFR	Methylenetetrahydrofolate Reductase
139.	P01374	LTA	Lymphotoxin Alpha
140.	O60602	TLR5	Toll Like Receptor 5
141.	P08581	MET	MET Proto-Oncogene, Receptor Tyrosine Kinase
142.	P42336	PIK3CA	Phosphatidylinositol-4,5-Bisphosphate 3-Kinase Catalytic Subunit Alpha
143.	O15263	DEFB4A	Defensin Beta 4A
144.	P04141	CSF2	Colony Stimulating Factor 2
145.	P12259	F5	Coagulation Factor V
146.	O15524	SOCS1	Suppressor Of Cytokine Signaling 1
147.	Q86YC2	PALB2	Partner And Localizer Of BRCA2
148.	P15692	VEGFA	Vascular Endothelial Growth Factor A
149.	Q14542	MIR126	MicroRNA 126
150.	P01116	KRAS	KRAS Proto-Oncogene, GTPase
151.	P01903	HLA-DRA	Major Histocompatibility Complex, Class II, DR Alpha
152.	O43852	MIR125A	MicroRNA 125a
153.	Q4FZB7	IL6-AS1	IL6 Antisense RNA 1
154.	Q86YH6	LOC126859963	BRD4-IndependentGroup4Enhancer GRCh37_chr7:22766269-22767468
155.	P43088	MIR223	MicroRNA 223
156.	P15884	TCF4	Transcription Factor 4
157.	Q9UKN1	MUC12	Mucin 12, Cell Surface Associated
158.	Q9NRD8	DUOX2	Dual Oxidase 2
159.	P42224	STAT1	Signal Transducer And Activator Of Transcription 1
160.	P08254	MMP3	Matrix Metallopeptidase 3
161.	Q9BQA9	CYBC1	Cytochrome B-245 Chaperone 1
162.	P29459	IL12A	Interleukin 12A
163.	P15260	IFNGR1	Interferon Gamma Receptor 1
164.	P0C0L4	C4A	Complement C4A (Chido/Rodgers Blood Group)
165.	Q03403	TFF2	Trefoil Factor 2
166.	P54707	ATP12A	ATPase H+/K+ Transporting Non-Gastric Alpha2 Subunit
167.	P11215	ITGAM	Integrin Subunit Alpha M
168.	P13726	F3	Coagulation Factor III, Tissue Factor
169.	Q9NPF7	IL23A	Interleukin 23 Subunit Alpha
170.	P06702	S100A9	S100 Calcium Binding Protein A9
171.	P09919	CSF3	Colony Stimulating Factor 3

172.	Q9P0L0	MIR142	MicroRNA 142
173.	Q15116	PDCD1	Programmed Cell Death 1
174.	P04439	HLA-A	Major Histocompatibility Complex, Class I, A
175.	P14174	MIF	Macrophage Migration Inhibitory Factor
176.	P32246	CCR1	C-C Motif Chemokine Receptor 1
177.	P01308	INS	Insulin
178.	P35520	CBS	Cystathionine Beta-Synthase
179.	Q29983	MICA	MHC Class I Polypeptide-Related Sequence A
180.	Q03426	MVK	Mevalonate Kinase
181.	Q15080	NCF4	Neutrophil Cytosolic Factor 4
182.	P38936	CDKN1A	Cyclin Dependent Kinase Inhibitor 1A
183.	Q9NZK5	ADA2	Adenosine Deaminase 2
184.	P40879	SLC26A3	Solute Carrier Family 26 Member 3
185.	P12724	RNASE3	Ribonuclease A Family Member 3
186.	Q13546	RIPK1	Receptor Interacting Serine/Threonine Kinase 1
187.	P58751	MIR146B	MicroRNA 146b
188.	Q9Y2G2	CARD8	Caspase Recruitment Domain Family Member 8
189.	P04155	TFF1	Trefoil Factor 1
190.	P01008	SERPINC1	Serpin Family C Member 1
191.	Q96PD4	IL17F	Interleukin 17F
192.	P24385	CCND1	Cyclin D1
193.	P10321	HLA-C	Major Histocompatibility Complex, Class I, C
194.	P98170	XIAP	X-Linked Inhibitor Of Apoptosis
195.	Q07812	BAX	BCL2 Associated X, Apoptosis Regulator
196.	P01127	PDGFB	Platelet Derived Growth Factor Subunit B
197.	P58753	TMX2-CTNND1	TMX2-CTNND1 Readthrough (NMD Candidate)
198.	P08311	CTSG	Cathepsin G
199.	Q9NRG4	LINC-ROR	Long Intergenic Non-Protein Coding RNA, Regulator Of Reprogramming
200.	P14210	HGF	Hepatocyte Growth Factor
201.	P29597	TYK2	Tyrosine Kinase 2
202.	P51671	CCL11	C-C Motif Chemokine Ligand 11
203.	P17706	PTPN2	Protein Tyrosine Phosphatase Non-Receptor Type 2
204.	Q9NZ08	ERAP1	Endoplasmic Reticulum Aminopeptidase 1
205.	P11926	ODC1	Ornithine Decarboxylase 1
206.	Q07654	TFF3	Trefoil Factor 3
207.	P20333	TNFRSF1B	TNF Receptor Superfamily Member 1B
208.	Q9HCK4	MIR195	MicroRNA 195
209.	P01033	TIMP1	TIMP Metallopeptidase Inhibitor 1
210.	P51684	CCR6	C-C Motif Chemokine Receptor 6

211.	P33076	CIITA	Class II Major Histocompatibility Complex Transactivator
212.	P02778	CXCL10	C-X-C Motif Chemokine Ligand 10
213.	P16871	IL7R	Interleukin 7 Receptor
214.	P08253	MMP2	Matrix Metallopeptidase 2
215.	P05107	ITGB2	Integrin Subunit Beta 2
216.	P07492	GRP	Gastrin Releasing Peptide
217.	P19320	VCAM1	Vascular Cell Adhesion Molecule 1
218.	P08729	KRT7	Keratin 7
219.	Q29980	MICB	MHC Class I Polypeptide-Related Sequence B
220.	Q13477	MADCAM1	Mucosal Vascular Addressin Cell Adhesion Molecule 1
221.	O95644	NFATC1	Nuclear Factor Of Activated T Cells 1
222.	P42768	WAS	WASP Actin Nucleation Promoting Factor
223.	P20366	TAC1	Tachykinin Precursor 1
224.	P05121	SERPINE1	Serpin Family E Member 1
225.	P10451	SPP1	Secreted Phosphoprotein 1
226.	P02749	APOH	Apolipoprotein H
227.	P60484	PTEN	Phosphatase And Tensin Homolog
228.	P08575	PTPRC	Protein Tyrosine Phosphatase Receptor Type C
229.	P80188	LCN2	Lipocalin 2
230.	Q9NXI6	RNF186	Ring Finger Protein 186
231.	Q9H3D4	TP63	Tumor Protein P63
232.	P21802	FGFR2	Fibroblast Growth Factor Receptor 2
233.	O43908	KLRC4	Killer Cell Lectin Like Receptor C4
234.	P00738	HP	Haptoglobin
235.	P00747	PLG	Plasminogen
236.	P00441	SOD1	Superoxide Dismutase 1
237.	P04626	ERBB2	Erb-B2 Receptor Tyrosine Kinase 2
238.	P06307	CCK	Cholecystokinin
239.	P29466	CASP1	Caspase 1
240.	Q8TAU0	NKX2-3	NK2 Homeobox 3
241.	Q16549	MIR141	MicroRNA 141
242.	P16581	SELE	Selectin E
243.	P09341	CXCL1	C-X-C Motif Chemokine Ligand 1
244.	P21781	FGF7	Fibroblast Growth Factor 7
245.	P12821	ACE	Angiotensin I Converting Enzyme
246.	P09683	SCT	Secretin
247.	Q96KG7	CYLD-AS1	CYLD Antisense RNA 1
248.	P98088	MUC5AC	Mucin 5AC, Oligomeric Mucus/Gel-Forming
249.	P12955	PEPD	Peptidase D

250.	P09601	HMOX1	Heme Oxygenase 1
251.	P08684	CYP3A4	Cytochrome P450 Family 3 Subfamily A Member 4
252.	Q9NR96	TLR9	Toll Like Receptor 9
253.	Q9UHD0	IL19	Interleukin 19
254.	P51681	CCR5	C-C Motif Chemokine Receptor 5
255.	Q9UGI6	MIR192	MicroRNA 192
256.	P00450	CP	Ceruloplasmin
257.	P47989	XDH	Xanthine Dehydrogenase
258.	Q8TDM6	DLG5	Discs Large MAGUK Scaffold Protein 5
259.	Q02641	NEAT1	Nuclear Paraspeckle Assembly Transcript 1
260.	P63000	RAC1	Rac Family Small GTPase 1
261.	O14543	SOCS3	Suppressor Of Cytokine Signaling 3
262.	P35408	PTGER4	Prostaglandin E Receptor 4
263.	P11226	MBL2	Mannose Binding Lectin 2
264.	P33681	CD80	CD80 Molecule
265.	Q96F07	MIR24-1	MicroRNA 24-1
266.	P10415	BCL2	BCL2 Apoptosis Regulator
267.	P41235	HNF4A	Hepatocyte Nuclear Factor 4 Alpha
268.	O14493	CLDN4	Claudin 4
269.	P42389	GAS5	Growth Arrest Specific 5
270.	P28340	POLD1	DNA Polymerase Delta 1, Catalytic Subunit
271.	A0A6I8PU40	TUG1	Taurine Up-Regulated 1
272.	P47923	MIR145	MicroRNA 145
273.	P15941	MUC1	Mucin 1, Cell Surface Associated
274.	Q5T2D3	OTUD3	OTU Deubiquitinase 3
275.	P17213	BPI	Bactericidal Permeability Increasing Protein
276.	P17211	MALAT1	Metastasis Associated Lung Adenocarcinoma Transcript 1
277.	P09211	GSTP1	Glutathione S-Transferase Pi 1
278.	P56856	CLDN18	Claudin 18
279.	P07949	RET	Ret Proto-Oncogene
280.	P12313	MIR221	MicroRNA 221
281.	Q8NBM4	UBAC2	UBA Domain Containing 2
282.	P17931	LGALS3	Galectin 3
283.	Q99626	CDX2	Caudal Type Homeobox 2
284.	O43353	RIPK2	Receptor Interacting Serine/Threonine Kinase 2
285.	Q99621	MIR146A	MicroRNA 146a
286.	P46527	CDKN1B	Cyclin Dependent Kinase Inhibitor 1B
287.	Q99346	MIR214	MicroRNA 214
288.	P22607	FGFR3	Fibroblast Growth Factor Receptor 3

289.	Q92326	MEG3	Maternally Expressed 3
290.	P01275	GCG	Glucagon
291.	P11912	CD79A	CD79a Molecule
292.	P20809	IL11	Interleukin 11
293.	P23458	JAK1	Janus Kinase 1
294.	Q9BYV9	BACH2	BTB Domain And CNC Homolog 2
295.	O95760	IL33	Interleukin 33
296.	Q06124	PTPN11	Protein Tyrosine Phosphatase Non-Receptor Type 11
297.	Q16873	MIR200C	MicroRNA 200c
298.	P04275	VWF	Von Willebrand Factor
299.	P11245	NAT2	N-Acetyltransferase 2
300.	O75015	FCGR3B	Fc Gamma Receptor IIIb
301.	P01135	TGFA	Transforming Growth Factor Alpha
302.	P18084	MIR215	MicroRNA 215
303.	P01138	NGF	Nerve Growth Factor
304.	P50851	LRBA	LPS Responsive Beige-Like Anchor Protein
305.	Q10981	FUT2	Fucosyltransferase 2
306.	Q01523	DEFA5	Defensin Alpha 5
307.	Q6F5E8	CARMIL2	Capping Protein Regulator And Myosin 1 Linker 2
308.	P10635	CYP2D6	Cytochrome P450 Family 2 Subfamily D Member 6
309.	P36544	MIR143	MicroRNA 143
310.	P40933	IL15	Interleukin 15
311.	P05019	IGF1	Insulin Like Growth Factor 1
312.	Q3MIT2	PUS10	Pseudouridine Synthase 10
313.	P09488	GSTM1	Glutathione S-Transferase Mu 1
314.	P78556	CCL20	C-C Motif Chemokine Ligand 20
315.	Q8TAX9	GSDMB	Gasdermin B
316.	P01344	IGF2	Insulin Like Growth Factor 2
317.	P11678	EPX	Eosinophil Peroxidase
318.	P15291	MIR320A	MicroRNA 320a
319.	P07942	LAMB1	Laminin Subunit Beta 1
320.	Q9P0X4	MIR139	MicroRNA 139
321.	P55290	MIR34A	MicroRNA 34a
322.	Q14832	BDNF-AS	BDNF Antisense RNA
323.	P0CG47	MIR29A	MicroRNA 29a
324.	P08637	FCGR3A	Fc Gamma Receptor IIIa
325.	Q9UBU3	GHRL	Ghrelin And Obestatin Prepropeptide
326.	P04629	NTRK1	Neurotrophic Receptor Tyrosine Kinase 1
327.	P01282	VIP	Vasoactive Intestinal Peptide

328.	Q13315	ATM	ATM Serine/Threonine Kinase
329.	P25942	CD40	CD40 Molecule
330.	Q9NPH9	IL26	Interleukin 26
331.	P23381	MIR140	MicroRNA 140
332.	Q9NP60	IL1RAPL2	Interleukin 1 Receptor Accessory Protein Like 2
333.	P00390	GSR	Glutathione-Disulfide Reductase
334.	Q9NZ71	RTEL1	Regulator Of Telomere Elongation Helicase 1
335.	Q99607	ELF4	E74 Like ETS Transcription Factor 4
336.	O15520	FGF10	Fibroblast Growth Factor 10
337.	P01112	HRAS	HRas Proto-Oncogene, GTPase
338.	O75469	NR1I2	Nuclear Receptor Subfamily 1 Group I Member 2
339.	P13612	ITGA4	Integrin Subunit Alpha 4
340.	Q92854	SEMA4D	Semaphorin 4D
341.	Q16665	HIF1A	Hypoxia Inducible Factor 1 Subunit Alpha
342.	P12872	MLN	Motilin
343.	P35900	KRT20	Keratin 20
344.	P08174	CD55	CD55 Molecule (Cromer Blood Group)
345.	P55085	F2RL1	F2R Like Trypsin Receptor 1
346.	P42658	SOD2-OT1	SOD2 Overlapping Transcript 1
347.	P16422	EPCAM	Epithelial Cell Adhesion Molecule
348.	P09622	DLD	Dihydrolipoamide Dehydrogenase
349.	Q12860	MIR18A	MicroRNA 18a
350.	P14598	NCF1	Neutrophil Cytosolic Factor 1
351.	P09917	ALOX5	Arachidonate 5-Lipoxygenase
352.	Q13761	RUNX3	RUNX Family Transcription Factor 3
353.	P61586	RHOA	Ras Homolog Family Member A
354.	P42830	CXCL5	C-X-C Motif Chemokine Ligand 5
355.	O43826	SLC37A4	Solute Carrier Family 37 Member 4
356.	P20701	ITGAL	Integrin Subunit Alpha L
357.	O14647	HLA-DRB9	Major Histocompatibility Complex, Class II, DR Beta 9 (Pseudogene)
358.	Q9Y6Q6	SNHG5	Small Nucleolar RNA Host Gene 5
359.	P25025	CXCR2	C-X-C Motif Chemokine Receptor 2
360.	Q9NZK7	PLA2G2E	Phospholipase A2 Group IIE
361.	Q9NQC7	CYLD	CYLD Lysine 63 Deubiquitinase
362.	P16671	CD36	CD36 Molecule
363.	P00813	ADA	Adenosine Deaminase
364.	Q09472	EP300	E1A Binding Protein P300
365.	Q9BQL6	FERMT1	FERM Domain Containing Kindlin 1
366.	P04179	SOD2	Superoxide Dismutase 2

367.	P11712	CYP2C9	Cytochrome P450 Family 2 Subfamily C Member 9
368.	Q07075	CBR3-AS1	CBR3 Antisense RNA 1
369.	O14746	TERT	Telomerase Reverse Transcriptase
370.	P14555	PLA2G2A	Phospholipase A2 Group IIA
371.	P16109	SELP	Selectin P
372.	Q15878	MIR150	MicroRNA 150
373.	P11169	MIR19A	MicroRNA 19a
374.	P10809	HSPD1	Heat Shock Protein Family D (Hsp60) Member 1
375.	Q9C000	NLRP1	NLR Family Pyrin Domain Containing 1
376.	P25963	NFKBIA	NFKB Inhibitor Alpha
377.	P20702	ITGAX	Integrin Subunit Alpha X
378.	Q02388	COL7A1	Collagen Type VII Alpha 1 Chain
379.	Q99808	MIR34C	MicroRNA 34c
380.	P00488	F13A1	Coagulation Factor XIII A Chain
381.	P24752	MIR222	MicroRNA 222
382.	Q13393	MIR451A	MicroRNA 451a
383.	Q15109	AGER	Advanced Glycosylation End-Product Specific Receptor
384.	P48764	SLC9A3	Solute Carrier Family 9 Member A3
385.	P22223	CDH3	Cadherin 3
386.	Q05655	PRKCD	Protein Kinase C Delta
387.	P42081	CD86	CD86 Molecule
388.	O95407	TNFRSF6B	TNF Receptor Superfamily Member 6b
389.	P43403	ZAP70	Zeta Chain Of T Cell Receptor Associated Protein Kinase 70
390.	P09237	MMP7	Matrix Metallopeptidase 7
391.	P06881	CALCA	Calcitonin Related Polypeptide Alpha
392.	Q92902	HPS1	HPS1 Biogenesis Of Lysosomal Organelles Complex 3 Subunit 1
393.	Q9BXL7	CARD11	Caspase Recruitment Domain Family Member 11
394.	P13501	CCL5	C-C Motif Chemokine Ligand 5
395.	P19875	CXCL2	C-X-C Motif Chemokine Ligand 2
396.	Q9UPA5	BSN	Bassoon Presynaptic Cytomatrix Protein
397.	O00187	MASP2	MBL Associated Serine Protease 2
398.	P36222	CHI3L1	Chitinase 3 Like 1
399.	Q9UL17	TBX21	T-Box Transcription Factor 21
400.	P01189	POMC	Proopiomelanocortin
401.	P08887	IL6R	Interleukin 6 Receptor
402.	P61769	B2M	Beta-2-Microglobulin
403.	Q13627	XIST	X Inactive Specific Transcript
404.	Q03519	TAP2	Transporter 2, ATP Binding Cassette Subfamily B Member
405.	P60022	DEFB1	Defensin Beta 1

406.	Q9NZQ7	CD274	CD274 Molecule
407.	P24298	GPT	Glutamic--Pyruvic Transaminase
408.	P04150	NR3C1	Nuclear Receptor Subfamily 3 Group C Member 1
409.	P10147	CCL3	C-C Motif Chemokine Ligand 3
410.	Q16610	ECM1	Extracellular Matrix Protein 1
411.	P51587	BRCA2	BRCA2 DNA Repair Associated
412.	Q8NEV9	IL27	Interleukin 27
413.	P09382	LGALS1	Galectin 1
414.	Q6W4X9	MUC6	Mucin 6, Oligomeric Mucus/Gel-Forming
415.	Q13976	MIR200A	MicroRNA 200a
416.	P10082	PYY	Peptide YY
417.	P10721	KIT	KIT Proto-Oncogene, Receptor Tyrosine Kinase
418.	P31994	FCGR2B	Fc Gamma Receptor IIb
419.	Q92851	CASP10	Caspase 10
420.	Q02556	IRF8	Interferon Regulatory Factor 8
421.	P10914	IRF1	Interferon Regulatory Factor 1
422.	O94778	AQP8	Aquaporin 8
423.	Q13873	KCNQ1OT1	KCNQ1 Opposite Strand/Antisense Transcript 1
424.	P09603	CSF1	Colony Stimulating Factor 1
425.	Q9UIF7	MUTYH	MutY DNA Glycosylase
426.	P02818	BGLAP	Bone Gamma-Carboxyglutamate Protein
427.	Q76B58	BRINP3	BMP/Retinoic Acid Inducible Neural Specific 3
428.	P00749	PLAU	Plasminogen Activator, Urokinase
429.	P10747	CD28	CD28 Molecule
430.	Q9NQG7	HPS4	HPS4 Biogenesis Of Lysosomal Organelles Complex 3 Subunit 2
431.	Q12841	MIR191	MicroRNA 191
432.	P41159	LEP	Leptin
433.	O00203	AP3B1	Adaptor Related Protein Complex 3 Subunit Beta 1
434.	P09429	HMGB1	High Mobility Group Box 1
435.	P01009	SERPINA1	Serpin Family A Member 1
436.	O14656	TOR1A	Torsin Family 1 Member A
437.	O14522	MIR199A1	MicroRNA 199a-1
438.	Q96RI1	NR1H4	Nuclear Receptor Subfamily 1 Group H Member 4
439.	P25205	MIR22	MicroRNA 22
440.	Q9HAW4	MIR210	MicroRNA 210
441.	Q9BYX4	IFIH1	Interferon Induced With Helicase C Domain 1
442.	Q5VV42	CDKAL1	CDK5 Regulatory Subunit Associated Protein 1 Like 1
443.	Q9NR97	TLR8	Toll Like Receptor 8
444.	P46013	MKI67	Marker Of Proliferation Ki-67

445.	P20062	TCN2	Transcobalamin 2
446.	Q15025	TNIP1	TNFAIP3 Interacting Protein 1
447.	Q9UI10	MIR148A	MicroRNA 148a
448.	P28067	HLA-DMA	Major Histocompatibility Complex, Class II, DM Alpha
449.	P14151	SELL	Selectin L
450.	P29474	NOS3	Nitric Oxide Synthase 3
451.	P05305	EDN1	Endothelin 1
452.	P0C0L5	C4B	Complement C4B (Chido/Rodgers Blood Group)
453.	Q9NSU2	TREX1	Three Prime Repair Exonuclease 1
454.	P24844	MIR10B	MicroRNA 10b
455.	Q96QV1	MIR424	MicroRNA 424
456.	P14921	ETS1	ETS Proto-Oncogene 1, Transcription Factor
457.	P01563	IFNA2	Interferon Alpha 2
458.	Q02846	MIR422A	MicroRNA 422a
459.	P56159	MIR30A	MicroRNA 30a
460.	P30711	GSTT1	Glutathione S-Transferase Theta 1
461.	Q30154	HLA-DRB5	Major Histocompatibility Complex, Class II, DR Beta 5
462.	P42772	CDKN2B	Cyclin Dependent Kinase Inhibitor 2B
463.	P84996	GNAS	GNAS Complex Locus
464.	Q9NSI6	MIR494	MicroRNA 494
465.	O75037	KIF21B	Kinesin Family Member 21B
466.	P62256	MIR182	MicroRNA 182
467.	P34130	IFNAR2-IL10RB	IFNAR2-IL10RB Readthrough
468.	P25788	MIR15A	MicroRNA 15a
469.	P62256	MIR200B	MicroRNA 200b
470.	O60826	MIR193A	MicroRNA 193a
471.	Q53FZ2	ACSM3	Acyl-CoA Synthetase Medium Chain Family Member 3
472.	Q9ULT0	TTC7A	Tetratricopeptide Repeat Domain 7A
473.	P25024	CXCR1	C-X-C Motif Chemokine Receptor 1
474.	P22894	MMP8	Matrix Metallopeptidase 8
475.	P16455	MGMT	O-6-Methylguanine-DNA Methyltransferase
476.	Q02763	TEK	TEK Receptor Tyrosine Kinase
477.	Q8ND76	CCNY	Cyclin Y
478.	Q0VDD5	PVT1	Pvt1 Oncogene
479.	Q99895	LINC01475	Long Intergenic Non-Protein Coding RNA 1475
480.	P48643	CCT5	Chaperonin Containing TCP1 Subunit 5
481.	Q02750	MAP2K1	Mitogen-Activated Protein Kinase Kinase 1
482.	Q02505	MUC3A	Mucin 3A, Cell Surface Associated
483.	O60880	SH2D1A	SH2 Domain Containing 1A

484.	O95163	ELP1	Elongator Acetyltransferase Complex Subunit 1
485.	P01562	IFNA1	Interferon Alpha 1
486.	P13747	HLA-E	Major Histocompatibility Complex, Class I, E
487.	P41231	MIR296	MicroRNA 296
488.	P51948	MIR675	MicroRNA 675
489.	Q13753	LAMC2	Laminin Subunit Gamma 2
490.	P12931	SRC	SRC Proto-Oncogene, Non-Receptor Tyrosine Kinase
491.	P16070	CD44	CD44 Molecule (Indian Blood Group)
492.	P98172	MIRLET7I	MicroRNA Let-7i
493.	P35221	CTNNA1	Catenin Alpha 1
494.	Q9HB29	IL1RL2	Interleukin 1 Receptor Like 2
495.	P27930	IL1R2	Interleukin 1 Receptor Type 2
496.	Q7Z5P9	MUC19	Mucin 19, Oligomeric
497.	O15392	BIRC5	Baculoviral IAP Repeat Containing 5
498.	Q9Y6R7	MIR224	MicroRNA 224
499.	P03372	ESR1	Estrogen Receptor 1
500.	Q6X784	ZPBP2	Zona Pellucida Binding Protein 2
501.	P45452	MMP13	Matrix Metallopeptidase 13
502.	O15144	ARPC2	Actin Related Protein 2/3 Complex Subunit 2
503.	Q7RTY1	NORAD	Non-Coding RNA Activated By DNA Damage
504.	P01106	MYC	MYC Proto-Oncogene, BHLH Transcription Factor
505.	Q8N138	ORMDL3	ORMDL Sphingolipid Biosynthesis Regulator 3
506.	P02751	FN1	Fibronectin 1
507.	P48061	CXCL12	C-X-C Motif Chemokine Ligand 12
508.	P13498	CYBA	Cytochrome B-245 Alpha Chain
509.	P42574	CASP3	Caspase 3
510.	P1323	MIR342	MicroRNA 342
511.	P09619	PDGFRB	Platelet Derived Growth Factor Receptor Beta
512.	Q9NYK1	TLR7	Toll Like Receptor 7
513.	P09884	POLA1	DNA Polymerase Alpha 1, Catalytic Subunit
514.	P13421	MIR9-1	MicroRNA 9-1
515.	P33151	MIR27A	MicroRNA 27a
516.	Q14139	MIR122	MicroRNA 122
517.	P59044	NLRP6	NLR Family Pyrin Domain Containing 6
518.	Q92835	INPP5D	Inositol Polyphosphate-5-Phosphatase D
519.	P17693	HLA-G	Major Histocompatibility Complex, Class I, G
520.	P16885	PLCG2	Phospholipase C Gamma 2
521.	P28065	PSMB9	Proteasome 20S Subunit Beta 9

522.	Q07864	POLE	DNA Polymerase Epsilon, Catalytic Subunit
523.	O95999	BCL10	BCL10 Immune Signaling Adaptor
524.	Q9Y6J3	SMAD5-AS1	SMAD5 Antisense RNA 1
525.	Q07157	TJP1	Tight Junction Protein 1
526.	P01833	PIGR	Polymeric Immunoglobulin Receptor
527.	Q15833	STXBP2	Syntaxin Binding Protein 2
528.	Q14790	CASP8	Caspase 8
529.	Q03001	DST	Dystonin
530.	Q92956	TNFRSF14	TNF Receptor Superfamily Member 14
531.	P04233	MIR100	MicroRNA 100
532.	P54278	PMS2	PMS1 Homolog 2, Mismatch Repair System Component
533.	P01823	LOC126806658	BRD4-Independent Group 4 Enhancer GRCh37_chr3:41265899-41267098
534.	P08700	IL3	Interleukin 3
535.	P46531	NOTCH1	Notch Receptor 1
536.	P38570	ITGAE	Integrin Subunit Alpha E
537.	P51692	STAT5B	Signal Transducer And Activator Of Transcription 5B
538.	P25103	TACR1	Tachykinin Receptor 1
539.	O93499	MIR23A	MicroRNA 23a
540.	P51449	RORC	RAR Related Orphan Receptor C
541.		MIR345	MicroRNA 345
542.	P48736	PIK3CG	Phosphatidylinositol-4,5-Bisphosphate 3-Kinase Catalytic Subunit Gamma
543.	Q9UNQ0	ABCG2	ATP Binding Cassette Subfamily G Member 2 (JR Blood Group)
544.	O95599	MIR106A	MicroRNA 106a
545.	P07204	THBD	Thrombomodulin
546.	Q99665	IL12RB2	Interleukin 12 Receptor Subunit Beta 2
547.	Q9Y6W8	ICOS	Inducible T Cell Costimulator
548.	P17174	GOT1	Glutamic-Oxaloacetic Transaminase 1
549.		MIR106B	MicroRNA 106b
550.	Q9NPP4	NLRC4	NLR Family CARD Domain Containing 4
551.	P27986	PIK3R1	Phosphoinositide-3-Kinase Regulatory Subunit 1
552.	Q92793	CREBBP	CREB Binding Protein
553.	P27169	PON1	Paraoxonase 1
554.	P17123	MIR20A	MicroRNA 20a
555.	P01111	NRAS	NRAS Proto-Oncogene, GTPase
556.	Q92733	HNF1A-AS1	HNF1A Antisense RNA 1
557.	P35869	AHR	Aryl Hydrocarbon Receptor
558.	P31785	IL2RG	Interleukin 2 Receptor Subunit Gamma
559.	Q16625	OCLN	Occludin
560.	Q92393	PGR-AS1	PGR Antisense RNA 1

561.	Q99836	MYD88	MYD88 Innate Immune Signal Transduction Adaptor
562.	P08069	IGF1R	Insulin Like Growth Factor 1 Receptor
563.	Q12882	DPYD	Dihydropyrimidine Dehydrogenase
564.	Q99856	MIR26B	MicroRNA 26b
565.	P01588	EPO	Erythropoietin
566.	P01042	KNG1	Kininogen 1
567.	P14902	IDO1	Indoleamine 2,3-Dioxygenase 1
568.	P00751	CFB	Complement Factor B
569.	P28482	MAPK1	Mitogen-Activated Protein Kinase 1
570.	Q93436	PCAT1	Prostate Cancer Associated Transcript 1
571.	P22004	BMP6	Bone Morphogenetic Protein 6
572.	P20815	CYP3A5	Cytochrome P450 Family 3 Subfamily A Member 5
573.	Q14213	EBI3	Epstein-Barr Virus Induced 3
574.	P16902	MIR93	MicroRNA 93
575.	P38398	BRCA1	BRCA1 DNA Repair Associated
576.	P06748	NPM1	Nucleophosmin 1
577.	Q53GD3	SLC44A4	Solute Carrier Family 44 Member 4
578.	P21902	HOTAIR	HOX Transcript Antisense RNA
579.	P13987	CD59	CD59 Molecule (CD59 Blood Group)
580.	P10144	GZMB	Granzyme B
581.	P14921	MIR204	MicroRNA 204
582.	P43116	PTGER2	Prostaglandin E Receptor 2
583.	P04440	HLA-DPB1	Major Histocompatibility Complex, Class II, DP Beta 1
584.	Q6N021	TET2	Tet Methylcytosine Dioxygenase 2
585.	P24855	DNASE1	Deoxyribonuclease 1
586.	Q99707	MTR	5-Methyltetrahydrofolate-Homocysteine Methyltransferase
587.	P34931	HSPA1L	Heat Shock Protein Family A (Hsp70) Member 1 Like
588.	Q6N023	MIR3936	MicroRNA 3936
589.	P18887	XRCC1	X-Ray Repair Cross Complementing 1
590.	O43918	AIRE	Autoimmune Regulator
591.	P38298	MIR423	MicroRNA 423
592.	P0DMV9	HSPA1B	Heat Shock Protein Family A (Hsp70) Member 1B
593.	P23398	MIR203A	MicroRNA 203a
594.	P16234	PDGFRA	Platelet Derived Growth Factor Receptor Alpha
595.	Q07817	BCL2L1	BCL2 Like 1
596.	P28023	MIR24-2	MicroRNA 24-2
597.	Q2LD37	BLTP1	Bridge-Like Lipid Transfer Protein Family Member 1
598.	P28062	PSMB8	Proteasome 20S Subunit Beta 8
599.	P98082	DAB2	DAB Adaptor Protein 2

600.	P12004	PCNA	Proliferating Cell Nuclear Antigen
601.	P28023	LOC130064510	ATAC-STARR-Seq Lymphoblastoid Silent Region 10661
602.	P16144	ITGB4	Integrin Subunit Beta 4
603.	Q70J99	UNC13D	Unc-13 Homolog D
604.	Q6P123	LOC106029312	Williams-Beuren Syndrome Medial Block B Recombination Region
605.	P14222	PRF1	Perforin 1
606.	P05412	JUN	Jun Proto-Oncogene, AP-1 Transcription Factor Subunit
607.	P31645	SLC6A4	Solute Carrier Family 6 Member 4
608.	Q6P179	ERAP2	Endoplasmic Reticulum Aminopeptidase 2
609.	O75473	LGR5	Leucine Rich Repeat Containing G Protein-Coupled Receptor 5
610.	Q14191	WRN	WRN RecQ Like Helicase
611.	Q16539	MAPK14	Mitogen-Activated Protein Kinase 14
612.	P32942	ICAM3	Intercellular Adhesion Molecule 3
613.	Q93038	TNFRSF25	TNF Receptor Superfamily Member 25
614.	Q6P172	MIRLET7E	MicroRNA Let-7e
615.	P49682	CXCR3	C-X-C Motif Chemokine Receptor 3
616.	Q6ZVW7	IL17REL	Interleukin 17 Receptor E Like
617.	Q07324	MIR30E	MicroRNA 30e
618.	Q86VZ6	JAZF1	JAZF Zinc Finger 1
619.	Q07321	CYTOR	Cytoskeleton Regulator RNA
620.	Q8TDQ0	HAVCR2	Hepatitis A Virus Cellular Receptor 2
621.	Q99075	HBEGF	Heparin Binding EGF Like Growth Factor
622.	Q07325	CXCL9	C-X-C Motif Chemokine Ligand 9
623.	Q07820	MCL1	MCL1 Apoptosis Regulator, BCL2 Family Member
624.	P02487	MROH3P	Maestro Heat Like Repeat Family Member 3, Pseudogene
625.	P18440	NAT1	N-Acetyltransferase 1
626.	O95267	RASGRP1	RAS Guanyl Releasing Protein 1
627.	P02787	MIR375	MicroRNA 375
628.	Q9H6D8	FNDC4	Fibronectin Type III Domain Containing 4
629.	Q04912	MST1R	Macrophage Stimulating 1 Receptor
630.	P29279	CCN2	Cellular Communication Network Factor 2
631.	Q13422	IKZF1	IKAROS Family Zinc Finger 1
632.	P15559	NQO1	NAD(P)H Quinone Dehydrogenase 1
633.	Q9Y581	INSL6	Insulin Like 6
634.	Q8NDB2	BANK1	B Cell Scaffold Protein With Ankyrin Repeats 1
635.	P00395	MT-CO1	Mitochondrially Encoded Cytochrome C Oxidase I
636.	P02781	RMRP	RNA Component Of Mitochondrial RNA Processing Endoribonuclease
637.	Q9BXN2	CLEC7A	C-Type Lectin Domain Containing 7A
638.	P02787	TF	Transferrin

639.	P13569	CFTR	CF Transmembrane Conductance Regulator
640.	P0CAT3	TLX1NB	TLX1 Neighbor
641.	P11586	MTHFD1	Methylenetetrahydrofolate Dehydrogenase, Cyclohydrolase And Formyltetra-hydrofolate Synthetase 1
642.	Q03518	TAP1	Transporter 1, ATP Binding Cassette Subfamily B Member
643.	O15455	TLR3	Toll Like Receptor 3
644.	Q8TD31	CCHCR1	Coiled-Coil Alpha-Helical Rod Protein 1
645.	O15519	CFLAR	CASP8 And FADD Like Apoptosis Regulator
646.	Q96F46	IL17RA	Interleukin 17 Receptor A
647.	P49913	CAMP	Cathelicidin Antimicrobial Peptide
648.	Q9BX69	CARD6	Caspase Recruitment Domain Family Member 6
649.	O15533	TAPBP	TAP Binding Protein
650.	P60953	MIR15B	MicroRNA 15b
651.	P05783	KRT18	Keratin 18
652.	P05787	KRT8	Keratin 8
653.	P60953	CDC42	Cell Division Cycle 42
654.	Q9UBC1	NFKBIL1	NFKB Inhibitor Like 1
655.	Q9UPW6	SATB2	SATB Homeobox 2
656.	Q99466	NOTCH4	Notch Receptor 4
657.	P08670	VIM	Vimentin
658.	P23510	TNFSF4	TNF Superfamily Member 4
659.	Q9UBC2	NCF4-AS1	NCF4 Antisense RNA 1
660.	P01854	IGHE	Immunoglobulin Heavy Constant Epsilon
661.	O75626	PRDM1	PR/SET Domain 1
662.	P37173	TGFBR2	Transforming Growth Factor Beta Receptor 2
663.	P16035	TIMP2	TIMP Metallopeptidase Inhibitor 2
664.	Q13635	PTCH1	Patched 1
665.	Q15848	ADIPOQ	Adiponectin, C1Q And Collagen Domain Containing
666.	O14965	AURKA	Aurora Kinase A
667.	O00175	CCL24	C-C Motif Chemokine Ligand 24
668.	Q13324	CRHR2	Corticotropin Releasing Hormone Receptor 2
669.	P09238	MMP10	Matrix Metallopeptidase 10
670.	P16284	PECAM1	Platelet And Endothelial Cell Adhesion Molecule 1
671.	P80098	CCL7	C-C Motif Chemokine Ligand 7
672.	Q9Y2G9	SBNO2	Strawberry Notch Homolog 2
673.	Q969H0	FBXW7	F-Box And WD Repeat Domain Containing 7
674.	Q15796	SMAD2	SMAD Family Member 2
675.	O75144	ICOSLG	Inducible T Cell Costimulator Ligand
676.	P51451	BLK	BLK Proto-Oncogene, Src Family Tyrosine Kinase

Table 4. 98 overlapping targets between SLBZS and UC.

Serial number	ID	Gene.symbol	Description
1.	P01375	TNF	Tumor Necrosis Factor
2.	P05231	IL6	Interleukin 6
3.	P22301	AHR	Aryl Hydrocarbon Receptor
4.	P01584	CYP1A1	Cytochrome P450 Family 1 Subfamily A Member 1
5.	P01579	IFNG	Interferon Gamma
6.	P05164	AKT1	AKT Serine/Threonine Kinase 1
7.	P10145	CXCL8	C-X-C Motif Chemokine Ligand 8
8.	P08183	MMP9	Matrix Metallopeptidase 9
9.	P01137	TGFB1	Transforming Growth Factor Beta 1
10.	P60568	IL10	Interleukin 10
11.	P04637	TP53	Tumor Protein P53
12.	P02741	CRP	C-Reactive Protein
13.	P05112	IL4	Interleukin 4
14.	P35222	CYP2E2	Cytochrome P450 Family 1 Subfamily EMember 2
15.	P35354	PTGS2	Prostaglandin-Endoperoxide Synthase 2
16.	P40763	STAT3	Signal Transducer And Activator Of Transcription 3
17.	P35228	NOS2	Nitric Oxide Synthase 2
18.	P31749	MPO	Myeloperoxidase
19.	P47712	PLA2G4A	Phospholipase A2 Group IVA
20.	P37231	PPARG	Peroxisome Proliferator Activated Receptor Gamma
21.	P03956	MMP1	Matrix Metallopeptidase 1
22.	Q04206	RELA	RELA Proto-Oncogene, NF-KB Subunit
23.	P23219	PTGS1	Prostaglandin-Endoperoxide Synthase 1
24.	Q96P20	NLRP3	NLR Family Pyrin Domain Containing 3
25.	P01583	IL1A	Interleukin 1 Alpha
26.	O96017	CHEK2	Checkpoint Kinase 2
27.	P05362	ICAM1	Intercellular Adhesion Molecule 1
28.	P48023	FASLG	Fas Ligand
29.	P01133	EGF	Epidermal Growth Factor
30.	P04040	CAT	Catalase
31.	P43897	NFE2L2	NFE2 Like BZIP Transcription Factor 2
32.	P29965	CD40LG	CD40 Ligand
33.	P13500	CCL2	C-C Motif Chemokine Ligand 2
34.	P00533	EGFR	Epidermal Growth Factor Receptor
35.	P08581	MET	MET Proto-Oncogene, Receptor Tyrosine Kinase
36.	P15692	VEGFA	Vascular Endothelial Growth Factor A
37.	Q9NRD8	DUOX2	Dual Oxidase 2
38.	P42224	STAT1	Signal Transducer And Activator Of Transcription 1

39.	P08254	MMP3	Matrix Metallopeptidase 3
40.	P13726	F3	Coagulation Factor III, Tissue Factor
41.	Q9NPF7	IL23A	Interleukin 23 Subunit Alpha
42.	P38936	CDKN1A	Cyclin Dependent Kinase Inhibitor 1A
43.	P24385	CCND1	Cyclin D1
44.	P98170	XIAP	X-Linked Inhibitor Of Apoptosis
45.	Q07812	BAX	BCL2 Associated X, Apoptosis Regulator
46.	P11926	ODC1	Ornithine Decarboxylase 1
47.	P01033	TIMP1	TIMP Metallopeptidase Inhibitor 1
48.	P02778	CXCL10	C-X-C Motif Chemokine Ligand 10
49.	P08253	MMP2	Matrix Metallopeptidase 2
50.	P19320	VCAM1	Vascular Cell Adhesion Molecule 1
51.	O95644	NFATC1	Nuclear Factor Of Activated T Cells 1
52.	P05121	SERPINE1	Serpin Family E Member 1
53.	P10451	SPP1	Secreted Phosphoprotein 1
54.	P60484	PTEN	Phosphatase And Tensin Homolog
55.	P00441	SOD1	Superoxide Dismutase 1
56.	P04626	ERBB2	Erb-B2 Receptor Tyrosine Kinase 2
57.	P16581	SELE	Selectin E
58.	P09601	HMOX1	Heme Oxygenase 1
59.	P08684	CYP3A4	Cytochrome P450 Family 3 Subfamily A Member 4
60.	P47989	XDH	Xanthine Dehydrogenase
61.	P10415	BCL2	BCL2 Apoptosis Regulator
62.	O14493	CLDN4	Claudin 4
63.	P09211	GSTP1	Glutathione S-Transferase Pi 1
64.	P09488	GSTM1	Glutathione S-Transferase Mu 1
65.	P01344	IGF2	Insulin Like Growth Factor 2
66.	P00390	GSR	Glutathione-Disulfide Reductase
67.	O75469	NR1I2	Nuclear Receptor Subfamily 1 Group I Member 2
68.	Q16665	HIF1A	Hypoxia Inducible Factor 1 Subunit Alpha
69.	P14598	NCF1	Neutrophil Cytosolic Factor 1
70.	P09917	ALOX5	Arachidonate 5-Lipoxygenase
71.	P16671	CD36	CD36 Molecule
72.	P25963	NFKBIA	NFKB Inhibitor Alpha
73.	Q05655	PRKCD	Protein Kinase C Delta
74.	P09237	MMP7	Matrix Metallopeptidase 7
75.	P19875	CXCL2	C-X-C Motif Chemokine Ligand 2
76.	P10914	IRF1	Interferon Regulatory Factor 1
77.	P00749	PLAU	Plasminogen Activator, Urokinase

78.	P29474	NOS3	Nitric Oxide Synthase 3
79.	P05305	EDN1	Endothelin 1
80.	P22894	MMP8	Matrix Metallopeptidase 8
81.	O15392	BIRC5	Baculoviral IAP Repeat Containing 5
82.	P03372	ESR1	Estrogen Receptor 1
83.	P45452	MMP13	Matrix Metallopeptidase 13
84.	P01106	MYC	MYC Proto-Oncogene, BHLH Transcription Factor
85.	P02751	FN1	Fibronectin 1
86.	P42574	CASP3	Caspase 3
87.	Q14790	CASP8	Caspase 8
88.	P48736	PIK3CG	Phosphatidylinositol-4,5-Bisphosphate 3-Kinase Catalytic Subunit Gamma
89.	Q9UNQ0	ABCG2	ATP Binding Cassette Subfamily G Member 2 (JR Blood Group)
90.	P07204	THBD	Thrombomodulin
91.	P27169	PON1	Paraoxonase 1
92.	P35869	IL-3	Interleukin 3
93.	P28482	MAPK1	Mitogen-Activated Protein Kinase 1
94.	P12004	PCNA	Proliferating Cell Nuclear Antigen
95.	P05412	JUN	Jun Proto-Oncogene, AP-1 Transcription Factor Subunit
96.	P31645	SLC6A4	Solute Carrier Family 6 Member 4
97.	Q16539	MAPK14	Mitogen-Activated Protein Kinase 14
98.	Q07820	MCL1	MCL1 Apoptosis Regulator, BCL2 Family Member
